# A desirability index framework for the bioprospecting of carrots grown in the Andean region for fresh, feed, cosmetic and nutraceutical applications

**DOI:** 10.3389/fpls.2026.1794157

**Published:** 2026-06-05

**Authors:** Juan Camilo Henao-Rojas, Cindy Umbacia-Ramirez, Tatiana Rodriguez-Quiroz, Joaquin Guillermo Ramirez-Gil, Edison Osorio

**Affiliations:** 1 Centro de Investigación La Selva, Agrosavia, Rionegro, Colombia; 2 Grupo de Investigación en Sustancias Bioactivas, Facultad de Ciencias Farmacéuticas y Alimentarias, Universidad de Antioquia UdeA, Medellín, Colombia; 3 Laboratorio de Agrocomputación y Análisis Epidemiológico, Departamento de Agronomía, Facultad de Ciencias Agrarias, Universidad Nacional de Colombia, sede Bogotá, Bogotá, Colombia

**Keywords:** agrobiodiversity, cosmetic, feed, fresh, functional food, MCDM, multi-criteria decision-making, quality trait

## Abstract

**Introduction:**

Carrot agrobiodiversity encompasses a broad range of compositional, sensory, technological, and biofunctional attributes. However, its industrial valorization remains constrained by the lack of quantitative decision criteria capable of translating sector-specific quality priorities into operational specifications for raw-material selection. This study proposes an application-driven bioprospecting framework for Andean carrot materials by integrating multidimensional quality data into phenotypically coherent typologies and industry-tailored desirability indices.

**Methods:**

Fourteen carrot materials grown under uniform Andean agronomic conditions were characterized using physicochemical, nutritional, colorimetric, safety-related, and biofunctional variables. Multivariate clustering, including PCA, K-means, and hierarchical clustering, was combined with univariate inference to identify statistically supported phenotypic contrasts. A mathematical framework implementing four sector-specific desirability indices—fresh consumption, functional foods, wet pet feed, and natural cosmetics—was developed using weighted utility functions, min–max normalization, and Gaussian penalty terms.

**Results:**

The analysis resolved four contrasting phenotypic groups: an extreme purple phenotype with elevated phenolic content and antioxidant capacity; a pale group with reduced carotenoid levels; and two differentiated orange groups showing variation in pigment density, soluble solids, dry matter content, and morphometric uniformity. The industry-specific indices produced differentiated rankings across value chains, identifying 6KUR and 5BER as promising materials for fresh consumption, 13FLA for functional foods and natural cosmetics, and 9NAN, 13FLA, and 8NAN for wet pet feed. These results also revealed structural trade-offs, as no single genotype simultaneously maximized performance across all evaluated sectors.

**Discussion:**

Overall, the proposed cluster–index–valorization workflow provides a reproducible quantitative framework linking agrobiodiversity characterization with industry-oriented raw-material selection. The approach supports early-stage portfolio design, sourcing decisions, and bioprospecting strategies for agro-industrial value chains prior to product-scale validation, while offering a transferable decision-support logic for plant matrices with relevant intra-specific variability.

## 1 Introduction

Over the last decade, the global market for functional foods, natural ingredients, and plant-based cosmetics has become a major driver of innovation in the agri-food and personal care sectors (Tachie et al., 2023; Rashidinejad, 2024). This trend is associated with increasing consumer demand for natural, multifunctional, and sustainable ingredients capable of supporting health and well-being while reducing dependence on synthetic additives (Dini and Laneri, 2019). The global functional food market was valued at USD 329.65 billion in 2023 and is projected to reach USD 586.06 billion by 2030 (Grand View Research, 2024), while the cosmeceuticals market reached USD 67.1 billion in 2024 (Grand View Research, 2025a), and natural food colorants exceeded USD 2.2 billion in 2024 (Grand View Research, 2025b). Recent evidence from the carrot value chain further indicates that consumer perception and market trends are increasingly aligned with value-added applications based on differentiated quality, naturalness, functionality, and territorial origin (Ospina-Sánchez et al., 2026).

These market trends reflect a broader transition toward plant-derived ingredients that combine bioactivity, technological functionality, and circular bioeconomic valorization potential (Tachie et al., 2023; Rashidinejad, 2024). Plant matrices rich in bioactive phytochemicals are increasingly positioned as alternatives to synthetic ingredients for greener and safer formulations (Michalak, 2023; Bandyopadhyay et al., 2025). However, their industrial use requires not only functional performance but also compliance with quality and safety requirements. In this context, microbiological, chemical, and physical hazards must be effectively controlled, as deviations can compromise consumer safety, technological stability, regulatory approval, commercial viability, and credibility of the final product (Van Boxstael et al., 2013).

Carrot (*Daucus carota* L.) is strategically important because of its extensive phenotypic diversity, global production scale, and distinctive phytochemical composition (Ospina-Sánchez et al., 2025; Martínez-Saldarriaga et al., 2026). China dominates global production with 44.5% of fresh carrot output, exceeding 18 million tons annually (Food and Agriculture Organization of the United Nations and World Health Organization, 2023; AtlasBig, 2023), and is associated with a robust innovation ecosystem for ingredient development and by-product valorization (Ospina-Sánchez et al., 2025). More broadly, Asia leads patent generation in agri-food technologies, with China accounting for nearly half of worldwide applications in 2023 (World Intellectual Property Organization, 2024), while Germany and the Netherlands emerge as key innovation hubs in Europe (European Patent Office, 2024).

The industrial versatility of carrot is strongly linked to genetic variability in pigment composition, which drives differences in color, antioxidant capacity, nutritional value, and health-related properties (Ospina-Sánchez et al., 2025; Kalia and Singh, 2023). Orange carrots are rich in α- and β-carotene and represent a major dietary source of provitamin A (Akram et al., 2021). Yellow carrots predominantly accumulate lutein, red carrots contain lycopene linked to cardiovascular health, purple and black carrots contain high levels of anthocyanins, and white carrots exhibit negligible pigment content (Da Silva, 2014; Dias, 2012; Sun et al., 2009; Singh et al., 2021). Purple genotypes often display higher bioactive concentrations than conventional orange cultivars, supporting their potential for nutraceutical and cosmetic applications (Pérez et al., 2023). In parallel, emerging technologies such as UVC irradiation and ultrasound-assisted extraction have expanded opportunities for the efficient valorization of carrot-derived by-products (Sánchez-Rangel et al., 2021; Carvajal et al., 2026).

Despite this potential, industrial carrot utilization remains inefficient. A substantial proportion of global carrot production fails to meet fresh-market standards, yet downgraded roots may retain carotenoids, phenolics, polyacetylenes, and ascorbic acid with potential value for cosmetics, nutraceuticals, and pet feed (Ahmad et al., 2019; Goyal et al., 2022; Michalak, 2023). However, utilization remains limited by the absence of standardized, multidimensional quality criteria for allocating raw materials to specific value chains (Jahanbakhshi et al., 2018). Current evaluation practices rely mainly on visual inspection and basic physicochemical parameters, which are insufficient to capture the nutritional, functional, safety-related, and technological potential of materials intended for animal feed, cosmetics, nutraceuticals, or functional foods (Amin et al., 2023; Quintero-Quiroz et al., 2026). Therefore, multidimensional characterization integrating morphological, physicochemical, nutritional, functional, colorimetric, and safety attributes is essential to define benchmarks and align carrot materials and by-products with emerging sector requirements (Ospina-Sánchez et al., 2025).

This challenge is particularly relevant in the Colombian Andean region, where genetic and phenotypic diversity in carrot represents a strategic asset for generating differentiated quality attributes (Moreno-Rodríguez et al., 2025). In tropical environments, interactions among genotype, altitude, soil properties, and climate can produce distinct multidimensional quality profiles (Ramírez-Gil et al., 2019; Horacio López-Hoyos et al., 2024). Valorization of non-commercial roots and processing by-products can reduce losses, improve resource efficiency, and enhance sustainability (Mrithula Mahalakshmi et al., 2025). In addition, aligning diversity-driven quality traits with industry-specific demands can support circular economy models and strengthen rural value chains (Kausar et al., 2025; Lizundia et al., 2022).

This study addresses this gap by performing a comprehensive multidimensional characterization of fourteen carrot materials, including white, yellow, orange, and purple phenotypes, cultivated under Andean tropical conditions. Thirty variables were evaluated, including morphological traits, bioactive compounds, antioxidant capacity, physicochemical parameters, nutritional composition, color attributes, and food safety indicators. By integrating these datasets, this study identifies differentiated quality profiles and proposes use-oriented desirability indices to support raw-material selection for fresh consumption, functional foods, pet feed, and natural cosmetics.

## 2 Material and methods

### 2.1 Plant material and experimental design

The plant material comprised fourteen carrot *(Daucus carota*) accessions representative of the genetic and phenotypic diversity currently cultivated commercially in the Colombian Andean region. Materials were coded as 1NAN, 2CHAN, 3CHAN, 4CHAN, 5BER, 6KUR, 7NAN, 8NAN, 9NAN, 10BER, 11NAN, 12NAN, 13FLA, and 14BER, representing predominantly orange-rooted phenotypes (11 accessions) and non-orange roots: white (11NAN), yellow (12NAN), and purple (13FLA). All materials were grown under open-field conditions at La Selva Research Center, AGROSAVIA (6°08′06″ N, 75°25′03″ W; 2,120 m a.s.l., Rionegro, Antioquia, Colombia), within the lower montane humid forest life zone (bh-MB; mean annual temperature 17 °C; mean relative humidity 78%). Crops were established on Andisol soils in raised beds (1.20 m × 5.00 m × 0.30 m) arranged in a randomized complete block design with four replicates per material.

Uniform agronomic management (including fertilization, supplemental irrigation, and integrated pest and disease control) was applied according to standardized protocols to minimize management-driven variability. Seeds were manually sown at 0.5 cm with 10 cm plant spacing and 15 cm between rows. Roots were harvested manually at commercial maturity from a central 1 m² subplot per bed to reduce border effects. Six independent subsamples per material were collected from each replicate, washed, morphologically verified, and immediately conditioned for subsequent physicochemical, nutritional and safety analyses (Castaño-Marín et al., 2026).

### 2.2 Characterization of multidimensional quality variables: physicochemical, nutritional, and functional parameters

#### 2.2.1 Physicochemical characterization

Physical parameters, including root weight, length, and diameter, were recorded for each sample. Color was assessed using a Konica Minolta CR-400 colorimeter following the CIE Lab* system for external and internal evaluations. Texture Profile Analysis (TPA) was performed with a TA-XT Plus texture analyzer on 23.8 mm cubes extracted at 70% of root length. Analyses comprised three randomly selected experimental units with three roots per material. For chemical analysis, 3 kg of carrot samples (excluding tips and peels) were homogenized. Moisture and dry matter content were determined gravimetrically per AOAC method 934.06 (Latimer, 2023b) by drying 2 g of sample at 105 °C. pH was measured using an Ohaus ST2100 pH meter according to AOAC method 981.12 (Latimer, 2023d), while total soluble solids were determined with a digital refractometer per AOAC method 932.12 (Latimer, 2023a), using juice from the homogenized carrots.

#### 2.2.2 Nutritional composition analysis

Nutritional profiling was conducted per the Colombian Ministry of Health’s Resolution 810 of 2021 (nutritional labeling standards). Analyzed parameters included total fat, sugars, dietary fiber, protein, carbohydrates, energy, ash content, and minerals, using validated Colombian Technical Standards. Total fat was assessed via Soxhlet extraction with hexane solvent (NTC 6240:2017; ICONTEC, 2017). Total sugars were measured using ultra-high-performance liquid chromatography (AOAC method 2018.16; Latimer, 2023c). Dietary fiber was evaluated with enzymatic-gravimetric techniques according to NTC 6383:2020 (ICONTEC, 2020) and AOAC method 991.43 (Latimer, 2023c). Protein was quantified using the Kjeldahl method based on nitrogen measurement (NTC 4657:2022; ICONTEC, 2022). Ash content was determined through gravimetric calcination at 550 °C (NTC-ISO 2171:2021; ICONTEC, 2021). Carbohydrates were calculated by difference, and energy value was determined using Atwater conversion factors accounting for macronutrient digestibility. Mineral elements were measured via atomic absorption spectrometry (NTC-EN 14084:2021 and NTC 5151:2003; ICONTEC, 2021 and 2003), which establishes protocols for mineral analysis in food.

#### 2.2.3 Functional potential and antioxidant capacity

Ethanolic extracts were prepared to evaluate the functional potential. Freeze-dried samples (200 mg) were extracted with 4 mL of 70% (v/v) ethanol, vortexed (5 min), sonicated (45 min), and centrifuged; the supernatant was brought to 5 mL.

Total phenolic content (TPC) was quantified by the Folin–Ciocalteu assay using a microplate reader (BioTek Epoch): 25 µL extract + 125 µL 10% Folin–Ciocalteu reagent + 100 µL sodium carbonate solution, incubated for 30 min, and read at 765 nm. Results were expressed as gallic acid equivalents using a calibration curve (Singleton and Rossi, 1965; Henao-Rojas et al., 2022).

FRAP was measured by mixing 10 µL extract with 250 µL FRAP working solution (acetate buffer, TPTZ, and FeCl_3_), incubating 30 min, and reading at 593 nm (results expressed as Trolox equivalents; Benzie and Strain, 1996). DPPH scavenging activity was determined by combining 150 µL extract with 50 µL DPPH working solution in 96-well plates, incubating 30 min in the dark, and reading at 517 nm; results were reported as % inhibition and Trolox equivalents (Blois, 1958; Zapata-Vahos et al., 2023). Hydrophilic (ORAC-H) and lipophilic (ORAC-L) ORAC assays were conducted by spectrofluorometry using fluorescein as probe. ORAC-H used phosphate buffer, whereas ORAC-L used randomly methylated β-cyclodextrin (RMCD) in acetone:water (50:50, v/v). Fluorescence decay (Ex/Em 485/525 nm) was monitored for 120 min, and values were expressed as Trolox equivalents (Prior et al., 2003).

Total carotenoids were quantified spectrophotometrically by extracting 83 mg of freeze-dried carrot with acetone, followed by ultrasonication and centrifugation. Extracts were diluted with methanol (50:50, v/v) and read at 449 nm in a microplate reader (BioTek Epoch) (Biswas et al., 2011; Castaño et al., 2025).

β-Carotene was quantified by HPLC-DAD following ultrasound-assisted extraction with antioxidant protection. Freeze-dried carrot (50 mg) was extracted with HPLC-grade hexane (5 mL) and sonicated (80 kHz, 20 min). The residue was re-extracted with an additional 5 mL hexane, and pooled extracts adjusted to 10.0 mL. An aliquot (2.0 mL) was diluted to 90% (v/v) extract + 10% (v/v) hexane containing BHT (100 ppm), and centrifuged (13,000 rpm, 10 min, 12 °C). The supernatant (1.0 mL) was evaporated under vacuum (Centrivap, Labconco, MO, USA) and reconstituted in THF + BHT (100 ppm) per established antioxidant protocols for labile carotenoids.

Chromatography was performed on an Agilent 1200/1260 HPLC system (degasser, multigradient valve, quaternary pump G7111A, autosampler G1329A, column thermostat G1316A, DAD G1315D) equipped with a reverse-phase C30 column (Nomura Chemical Develosil C30-UG, 5 µm, 4.6 × 250 mm). The mobile phase consisted of IPA and an equimolar ACN: MeOH mixture (1:1), with all solvents conditioned with BHT (100 ppm) and TEA (500 ppm). Flow rate was 0.7 mL/min under the following gradient: 0–10 min, IPA 20–80% with ACN and MeOH 40–10% each; 10–28 min, 80% IPA/10% ACN/10% MeOH; 28–28.5 min, return to initial conditions; 28.5–30 min, re-equilibration at 20% IPA/40% ACN/40% MeOH. Injection volume was 5 µL, column temperature 18 °C, and quantification was at 450 nm (bandwidth 4 nm). External calibration used β-carotene standards in THF (BHT 100 ppm): a 100 ppm stock was prepared by dissolving 1.0 mg β-carotene in 10 mL THF, and working standards spanned the quantification range. Linearity was obtained over 1.56–50 µg/mL (r ≈ 0.9997–0.9998). Peaks were integrated in OpenChrom using automated first-derivative peak detection and trapezoidal area integration (default settings), and identity was confirmed by retention time and UV–Vis spectra (190–700 nm) versus the reference standard.

#### 2.2.4 Data analysis

Multivariate analyses integrated physicochemical, nutritional, functional, metabolomic, and sensory variables to reveal latent structure and statistically coherent sample groupings. Datasets were first checked for consistency and completeness; outliers were screened using distribution-based criteria and leverage diagnostics, and variables with extensive or non-random missingness were removed. All variables were mean-centered and autoscaled to unit variance (z-scores) to standardize contributions across measurement scales and satisfy variance- and distance-based assumptions.

Unsupervised PCA was used for exploratory dimensionality reduction, summarizing covariance into orthogonal components to identify dominant sources of variability, assess variable correlations, and visualize samples in latent space. Component retention was guided by explained variance and eigenvalue structure. Clustering was then performed on standardized data: hierarchical clustering was used to explore global similarity patterns, followed by K-means to refine membership by minimizing within-cluster variance. The number of clusters was selected by within-cluster inertia using the elbow criterion. Discriminant analyses were subsequently conducted using cluster membership as the grouping factor to quantify group separation and the contribution of individual variables to discrimination; classification performance was used to evaluate robustness and consistency of the multivariate structure.

For univariate inference, each physicochemical, nutritional, colorimetric, microbiological, and biofunctional variable was evaluated for normality and homoscedasticity (Shapiro–Wilk and Levene), followed by one-way ANOVA (α = 0.05) to test differences among materials and, in parallel, among clusters. When significant effects were observed, Tukey’s HSD was applied for multiple comparisons, reporting grouping letters to identify statistically equivalent levels and univariate extremes. Results were summarized in comparative visualizations based on standardized profiles (z-scores) to contrast trait magnitudes across varieties and cluster centroids and to support interpretation of actionable phenotypic “packages” and groups.

### 2.3 Determination of the basic characteristics of multidimensional quality factors in fresh consumption, animal feed, nutraceutical, and cosmetic industries

Quality criteria for carrot utilization across four industrial sectors (fresh consumption, animal feed, nutraceutical, and cosmetic) were defined using a combined approach: primary sectoral data collected directly from industry stakeholders (Moreno-Rodríguez et al., 2025) were triangulated with a systematic literature review of Scopus, Web of Science, and ScienceDirect. Predefined search queries were structured by application and combined terms related to: “*Daucus carota* L.”, “fresh market carrot quality”, “consumer acceptance attributes”, “root size uniformity”, “external color quality”, “CIELAB color parameters”, “soluble solids (°Brix)”, “industrial carrot processing”, “dry matter yield”, “carrot-derived functional foods”, “antioxidant capacity”, “phenolic compounds”, “carotenoids”, “nutraceutical carrot products”, “animal feed ingredients”, “pet food raw materials”, “dietary fiber functionality”, “carrot-based cosmetic ingredients”, “natural cosmetic actives”, “plant-derived antioxidants”.

Quality indicators were identified, weighted, and ranked using natural language processing (NLP) techniques that analyzed word frequency, contextual relevance, and co-occurrence patterns across sources to prioritize key multidimensional attributes (Ospina-Sánchez et al., 2025). The derived indicators were validated against documented sectoral requirements and value-chain profiles (Moreno-Rodríguez et al. (2025), ensuring alignment with the functional, technological, and market-driven needs of each industrial application.

### 2.4 Determination, validation, and application of industrial usability indices

Sector-specific usability indices were developed to summarize the multidimensional performance of the evaluated carrot varieties across fresh consumption, processed foods, pet feed, and natural cosmetics. To identify relevant variables for carrot-based industrial applications, Multicriteria Decision Analysis (MCDA) was implemented as a structured framework to integrate multiple, potentially conflicting criteria and to support objective prioritization (Belton and Stewart, 2002; Frej et al., 2017). This approach allows the simultaneous evaluation of quality, functional, technological, and market-oriented variables by assigning weights based on their relative importance to specific applications (Gésan-Guiziou et al., 2020; Belton and Stewart, 2002; Frej et al., 2017). MCDA is widely used in agri-food systems to support sustainability and value-chain optimization (Belton and Stewart, 2002; Gésan-Guiziou et al., 2020; Frej et al., 2017).

A multicriteria decision index (**Equation 1**) was applied to integrate and prioritize relevant variables. This industrial suitability index represents an overall measure of desirability for each evaluated alternative, calculated as a single normalized decision function D(x,y) that maps the two-dimensional space R^2^ onto the interval [0,1]. Values of D(x,y)=1 indicate maximum desirability or preference, whereas D(x,y)=0 represents a non-desirable alternative that does not meet the defined criteria. A value of D(x,y)=0.5 corresponds to an intermediate or neutral level of desirability, reflecting balanced performance across the evaluated attributes. Individual desirability functions dx(x) and dy(y) were first computed for each attribute independently, with values normalized to the range [0,1]. The global decision index D(x,y) was then obtained by integrating these individual desirability functions, providing a single comparable metric that supports objective evaluation and decision-making in contexts involving multiple industrial objectives.

The indices were then calculated as weighted linear combinations of these normalized variables, using sector-specific weighting coefficients that reflect the relative importance of each attribute within the corresponding industrial context. Specifically, in our process of **Equation 1**, we started from the assumption that 0.5 = 50% contribution; Min–max normalization=When x = min(x), the normalized value equals 0, whereas when x = max(x), corresponding to the best observed value, the normalized value equals 1 (**Equation 2**). In this approach, x represents a desirable variable (e.g., carotenoid content or soluble solids), whereas y represents an undesirable variable (e.g., microbial load or color deviation). Additionally, Gaussian penalty functions (**Equation 3**) were incorporated for variables whose suitability is associated with values near a defined optimum (e.g. sugar content for fresh market consumption), progressively penalizing deviations from the target value. The parameter σ is an adjustment factor that controls the intensity of the penalty associated with undesirable attributes and can be calculated based on the standard deviation of the undesirable variables. Both components are normalized to generate partial desirability scores ranging from 0 to 1. The resulting index values were then rescaled to a 0–100 point scale to facilitate the interpretation and cross-comparison of industrial use potential among carrot varieties. Consequently, the global decision index D(x,y) is constrained within the interval 0≤D(x,y)≤1, where higher values indicate greater overall desirability resulting from high levels of desirable attributes and low levels of undesirable ones.

For undesirable variables, a Gaussian penalty function was applied using the utility function described in **Equation 3** where this formulation allows the desirability score to decrease smoothly and nonlinearly as the magnitude of the undesirable attribute increases. The Gaussian structure penalizes deviations proportionally to their squared distance from the optimal condition (y≈0), making the index sensitive to large deviations while remaining robust to small fluctuations around low values. The parameter σ controls the intensity of the penalization and was fixed to provide a balanced trade-off between sensitivity and stability, ensuring comparability across samples. This approach is particularly appropriate for critical undesirable attributes, such as microbial load or color deviation, where extreme values must be strongly penalized. The resulting utility scores are bounded between 0 and 1, facilitating their integration into a multicriteria decision index while preserving statistical interpretability and reproducibility.

(1) $$D\left(x,y\right)=\sum_{i=1}^{k}w_{i}\;u_{i}\left(xi\right)+w_{g}G_{g}\left(y\right)\text{ with }\sum_{i=1}^{k}w_{i}+w_{g}=1$$

Decomposition of the index components

Where normalization of variable *x*:

For a variable x to be maximized, a min-max utility function was applied:

(2) $$u_{i}\left(xi\right)=\frac{xi-min\left(xi\right)}{(max\left(xi\right)-min\left(xi\right)},\text{ with }0\leq u_{i}\left(xi\right)\leq 1,$$

Where:

*xi*: represents the observed value of the quantitative variable i. These variables correspond to physicochemical, functional or bioactive attributes that are relevant for industrial evaluation; min (*xi*)and max 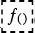 (*xi*) correspond to the minimum and maximum values observed in the global distribution of all evaluated varieties; u_i_ (*xi*): normalized utility of variable *x*_i_; *y:* represents the deviation of a variable or a set of variables from its target value, quantifying the degree of functional or technological imbalance relevant to the evaluated industrial application.

(3) $$\text{Gaussian penalty function: }G_{g}\left(y\right)=exp\left(-\frac{y^{2}}{2\sigma^{2}}\right),\text{ with }0\leq G_{g}\left(y\right)\leq 1;$$

Where:

σ = 50; G_g_ (y): Gaussian penalty function associated with deviation *y*; w*_i_*: relative weight of variable *x*_i_; w*_g_*: weight assigned to the Gaussian penalty term; σ: tolerance parameter controlling the strength of penalization.

### 2.5 Assessment of carrot genotype suitability for specific industrial uses based on an industrial desirability index

Each genotype was evaluated across industrial categories using the weighted industrial desirability framework described above. For each genotype–industry combination, weighted index values were visualized as heat maps to enable direct comparison of performance across sectors and to support industry-specific selection aligned with functional and quality requirements. To strengthen industrial decision-making, influential variables for each industry were examined using a quartile-based approach. For each key variable, genotype values were assigned to four global quartiles based on the empirical distribution across all genotypes, where Q4 denotes optimal performance and Q1 indicates less favorable values. This procedure supports indirect optimization by simultaneously identifying high-performing genotypes and the variables driving industrial suitability. All analyses were implemented in Python to ensure reproducibility. Statistical processing and segmentation relied on standard scientific libraries, and visualizations were generated with Plotly (interactive) and Matplotlib (static). The workflow was organized into sequential phases, as described below.

To convert physicochemical and functional measurements into interpretable indicators of industrial value, synthetic composite variables were derived: (i) Color Index (CI), computed to quantify chromatic intensity from CIELAB coordinates (**Equation 4**); and (ii) Antioxidant Index (AI), a weighted composite integrating four antioxidant assays to better capture antioxidant functionality than any single method, with weights assigned according to the analytical specificity and relevance of each technique (**Equation 5**).

(4) $$IC=\frac{C^{*}.a^{*}}{L}$$

(5) Antiox=0.35(ORAC)+0.25(FRAP)+0.25(DPPH)+0.15(TPC)

To reduce biological variability and intra-plot environmental effects, replicate-level measurements were aggregated into genotype centroids (arithmetic means). The resulting dataset included core performance variables (e.g., °Brix, dry matter, fresh weight, CI, AI) and additional industry-specific variables.

Each variable was segmented using global quartiles (Q1–Q4) computed across all genotypes, establishing competitive thresholds within the evaluated population and enabling standardized cross-industry comparisons. Accordingly, each genotype was assigned to a quartile level for every variable.

Final classification used a Dominant Quartile Algorithm, which assigns an overall performance category to each genotype based on the frequency with which it falls within a given quartile across the three most industry-relevant variables. For bimodal outcomes, the higher-performance quartile (Q4) was prioritized to ensure conservative selection favoring superior industrial potential. Industrial “quality spaces” were visualized as 3D Plotly collages, where each point represents a genotype centroid located in a space defined by three industry-relevant axes (e.g., °Brix, dry matter, and antioxidant index). Semi-transparent orthogonal planes at quartile boundaries (Q1–Q3) partitioned the space into discrete quality regions. Marker sizes were linearly scaled by the number of samples (n) per genotype to support visual appraisal of representativeness and robustness.

### 2.6 Food safety assessment and pesticide residue analysis

Food safety analyses were conducted in accordance with Resolution 2674 of 2013 issued by the Colombian Ministry of Health and Social Protection, which establishes sanitary requirements for food production, processing, storage, and commercialization. Microbiological parameters included total mesophilic aerobic count, total coliforms, fecal coliforms, *Salmonella* spp., molds, and yeasts, following validated NTC methodologies. *Mesophilic aerobes* were enumerated by plate count on Plate Count Agar (PCA) (30 °C, 48 h) according to NTC 3908:2025 (ICONTEC, 2025). Total coliforms were determined by membrane filtration on violet red bile agar (VRBA) (37 °C, 24 h) and fecal coliforms by the most probable number (MPN) method in EC broth at 44.5 °C following NTC 4458:2018 (ICONTEC, 2018). *Salmonella* spp. detection included pre-enrichment in buffered peptone water, selective enrichment, and isolation on XLD and HE agars NTC 4574:2007 (ICONTEC, 2007). Molds and yeasts were quantified by surface plating on potato dextrose agar (PDA) and incubation at 25 °C for 3–5 days, following NTC 5698-1:2009 (ICONTEC, 2009). Heavy metals (copper, nickel, cadmium, and lead) were quantified by flame atomic absorption spectrometry according to Resolución 4506 de 2013 and NTC-EN 14084:2021 (ICONTEC, 2021).

Pesticide residues were determined using a multi-residue GC/LC method after acetonitrile extraction and dispersive solid-phase extraction (d-SPE) clean-up, in accordance with method UNE-EN 15662:2019, (2019). Instrumental analyses complied with the standard’s performance criteria (calibration linearity, quality control samples, blanks, fortified samples, and matrix-specific detection limits) to ensure reliable determination of maximum residue limits in carrot samples.

Complementarily, a systematic review was conducted of the regulatory scope of national and international requirements applicable to carrot quality and safety across industrial uses. Searches in official regulatory repositories and institutional databases of international organizations, regulatory authorities, and standardization bodies used predefined keywords. Documents were screened for relevance, validity, and current applicability, then analyzed and classified by industrial sector, type of microbiological or chemical risk, and regulatory level.

## 3 Results

### 3.1 Multivariate structure and clustering of carrot materials based on multidimensional quality traits

The multivariate assessment of carrot (*Daucus carota* L.) materials, integrating physicochemical, nutritional, colorimetric, microbiological, safety-related, and biofunctional variables, revealed a structured intra-specific variability that can be parsimoniously summarized into four internally coherent groups. The optimal number of clusters was supported by the elbow criterion applied to the within-cluster sum of squares (inertia), which showed a clear inflection at *k* = 4, indicating that increasing *k* beyond this point would provide only marginal gains in within-group compactness relative to the added complexity (**Supplementary Figure 1**). The robustness of this four-group structure was further supported by the agreement between partition-based and hierarchical clustering strategies applied to the same standardized multidimensional dataset (**Supplementary Figure 2**). Specifically, the K-means solution (*k* = 4) produced compact partitions with consistent separation patterns, whereas agglomerative hierarchical clustering using Ward’s method generated a dendrogram topology that recapitulated the same macro-structure, characterized by high within-cluster homogeneity and pronounced between-cluster dissimilarity. The convergence between these conceptually distinct clustering paradigms provides methodological triangulation, supporting the four-cluster solution as a statistically defensible condensation of intra-specific diversity into groups with comparable multidimensional quality profiles.

**Figure 1:**
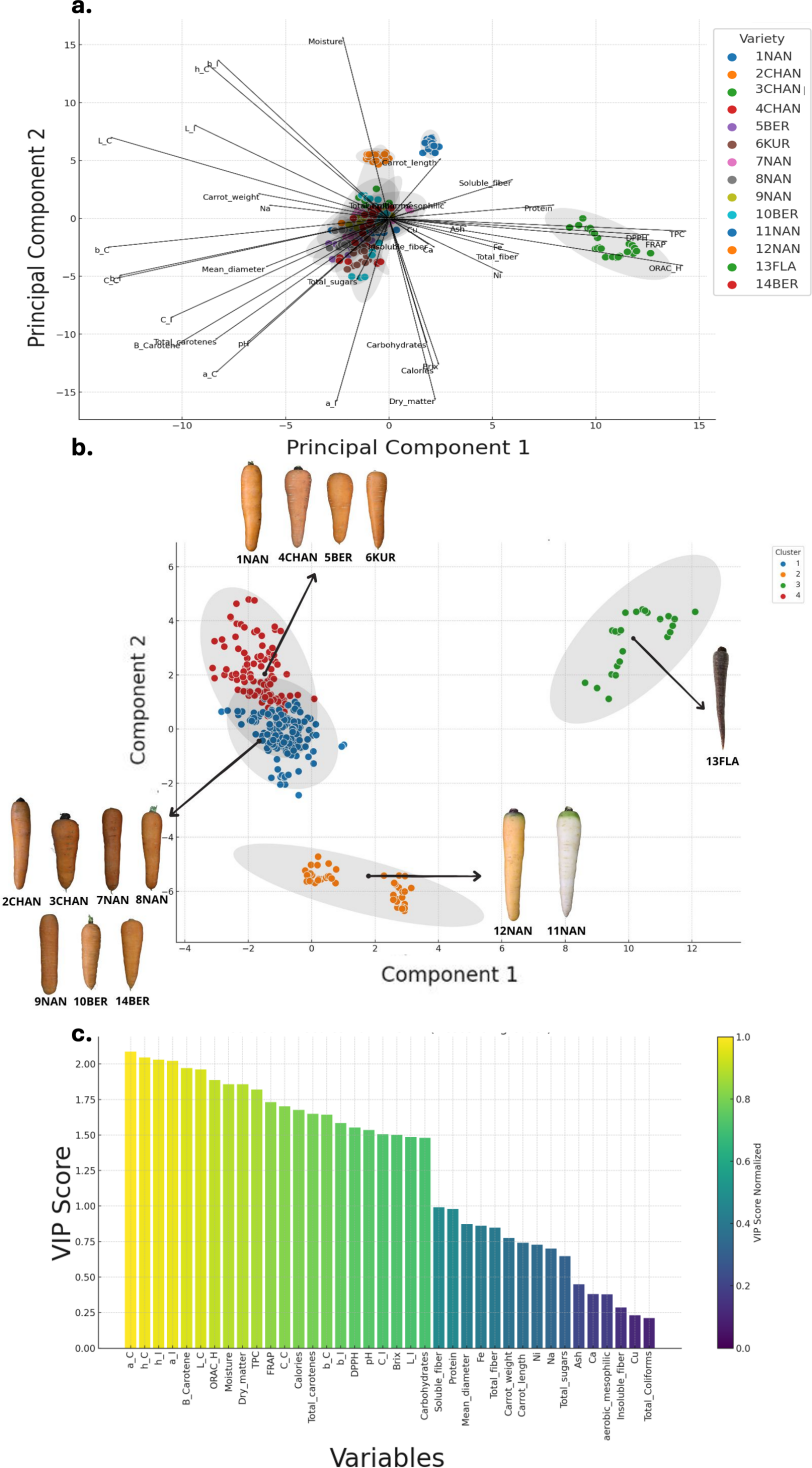
Multivariate discrimination of carrot (*Daucus carota* L.) materials: **(a)** PCA biplot of multidimensional traits, **(b)** cluster allocation and phenotypic grouping, and **(c)** PLS-DA VIP scores highlighting the most discriminant variables.

The PCA biplot evidenced a strongly structured multivariate space, revealing substantial intra-specific variability among the evaluated carrot materials and a clear organization of trait co-variation (**Figure 1a**). A central feature of the ordination was the presence of a highly displaced material, 13FLA, which appeared clearly separated from the bulk of samples along the major multivariate gradient. This separation aligned not only with colorimetric descriptors but also with vectors associated with biofunctional and antioxidant capacity, including TPC, FRAP, DPPH, and ORAC-H, indicating that 13FLA exhibits a markedly distinct functional phenotype characterized by consistently elevated antioxidant-related metrics relative to the remaining materials. The strong co-linearity of these antioxidant vectors further suggests that the multivariate distance of 13FLA is not attributable to a single endpoint, but rather to a coordinated shift in biofunctional attributes.

Importantly, the four-cluster structure should not be interpreted as a classification based exclusively on visual root color. Although colorimetric variables were among the strongest contributors to multivariate discrimination, as evidenced by the PCA biplot and the PLS-DA VIP ranking, cluster membership was obtained from the complete standardized multidimensional dataset. Thus, in this carrot panel, color emerged as a dominant phenotypic signal, but it operated in coordination with other industrially relevant trait blocks, including pigment-associated composition, antioxidant capacity, soluble solids, dry matter, moisture, morphometric traits, and safety-related variables. The purple material 13FLA, for instance, was separated not only by its chromatic coordinates but also by its high phenolic content and antioxidant capacity, whereas the pale materials were differentiated by reduced pigment-associated attributes and a distinct compositional profile. Likewise, the two orange groups differed in subtler but relevant variables such as pigment density, soluble solids, dry matter, root size, and morphometric uniformity. Consequently, the clusters are best understood as multidimensional quality typologies rather than simple color classes.

This interpretation is case-dependent and should not be generalized uncritically to other plant matrices or crop panels. In the present study, the broad phenotypic contrast among orange, pale, and purple carrots made color a highly informative discriminator of multidimensional quality variation. However, when this cluster-based framework is extrapolated to other biological matrices, the dominant drivers of group separation should be reassessed empirically, as clustering may depend on less visually apparent physicochemical, nutritional, safety-related, or biofunctional gradients. Therefore, the relevance of color as a discriminatory axis should be understood as an outcome of the specific carrot materials evaluated here, rather than as a predefined rule of the proposed bioprospecting framework.

Beyond this extreme material, the PCA also suggested additional, interpretable gradients that differentiate subsets of materials. Colorimetric traits, particularly those capturing chromaticity and hue in external and internal tissues (e.g., a_C, h_C, a_I, h_I, together with related CIELAB-derived coordinates) displayed large vector magnitudes and well-defined directions, evidencing that pigmentation-linked optical signatures represent a major source of variance in the dataset (**Figure 1a**). Notably, several orange-root materials clustered more tightly around the centroid region, reflecting shared or partially overlapping multivariate profiles when all trait blocks are jointly considered. This pattern indicates that, despite measurable univariate differences among orange materials, their overall multivariate dispersion is comparatively modest relative to the extreme displacement observed for 13FLA. In practical terms, the biplot supports the interpretation that most orange varieties express a broadly similar multivariate “baseline”, whereas materials with unusual pigmentation and/or functional chemistry can drive large-scale separations in PCA space.

A secondary gradient was associated with the water–solids balance, where moisture opposed variables related to solids accumulation (dry matter, carbohydrates and calories). This configuration reflects a compositional trade-off: samples with higher moisture content project toward the moisture vector, while those with greater solids density project toward dry matter and energy-related traits (**Figure 1a**). This axis provided a meaningful secondary structure to the dataset, contributing to separation among subsets of materials even when they remain closer than 13FLA to the centroid. Morphometric descriptors (carrot weight, carrot length, and mean diameter) showed intermediate loadings compared with the dominant biochemical and colorimetric gradients, indicating that size/shape contributes to dispersion but is not the primary determinant once compositional and functional traits are integrated.

**Figure 2:**
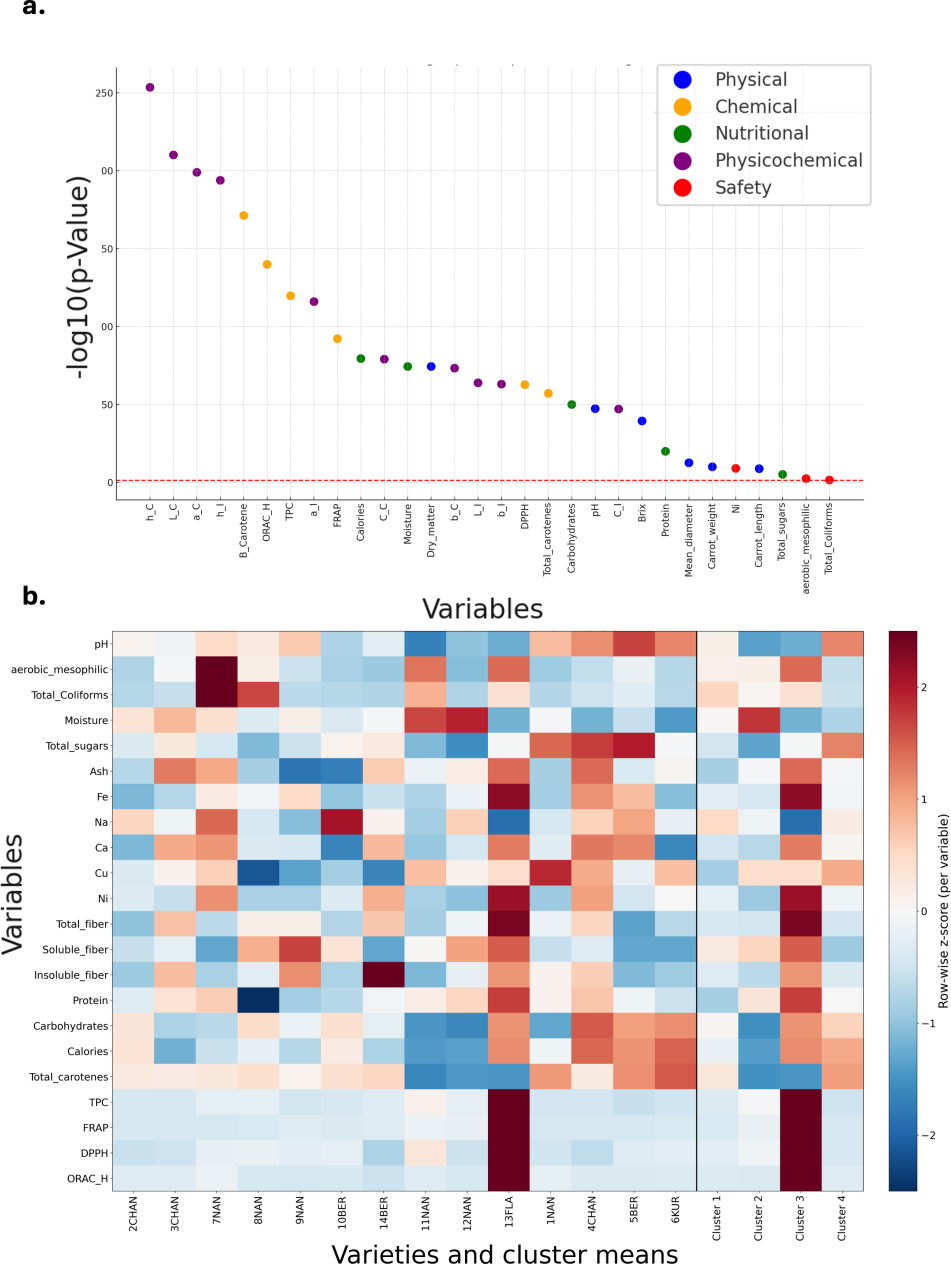
**(a)** Manhattan plot of significance (−log10 pvalue) for ANOVA contrasts between carrot materials studied. **(b)** Normalized heatmap of multidimensional quality traits in carrot (*Daucus carota* L.) varieties and multivariate clusters. mean values and Tukey’s HSD groupings. Different lowercase letters indicate significant differences among carrot varieties according to Tukey’s HSD test (α = 0.05). Different uppercase letters indicate significant differences among clusters generated at the multivariate level according to Tukey’s HSD test (α = 0.05).

Supervised PLS-DA was implemented to maximize between-group separation and identify variables with the highest discriminatory power (**Figures 1b, c**). The VIP profile indicated that colorimetric traits dominate the discriminant structure, with the highest VIP values concentrated in external and internal color parameters (notably a_C, h_C, h_I, a_I, and allied coordinates). This result provides strong statistical support for the notion that pigmentation-linked optical traits are not merely descriptive but function as high-leverage predictors of class membership in the supervised framework. In parallel, pigment-related compositional markers, particularly β-carotene and total carotenoids, ranked among the most influential variables, mechanistically bridging color expression with underlying carotenoid chemistry. Critically, the antioxidant block (TPC, FRAP, DPPH, ORAC_H) remained within the high-to-moderate VIP, corroborating the PCA interpretation and explaining why the most extreme multivariate displacement in the biplot is associated with the functional axis dominated by these metrics (**Figures 2a-c**). In contrast, several microbiological indicators and multiple mineral/trace elements tended to populate the lower VIP region, suggesting that, under the present dataset structure, they provide limited incremental power for discrimination compared with pigmentation and antioxidant functionality (**Figure 1c**).

### 3.2 Intraspecific and intercluster contrasts in physicochemical, nutritional, colorimetric, and biofunctional traits

The univariate analysis of variance (ANOVA) summarized in **Figure 2a** indicates that the vast majority of measured traits differed significantly among the carrot materials (comparison shown with reference line at p = 0.05), with particularly strong signals concentrated in the block of colorimetric variables (h_C, L_C, a_C) and in the biofunctional assessment criteria (ORAC_H, TPC, FRAP, DPPH). This pattern supports a highly structured phenotypic and functional diversity across the entire population of evaluated materials. In contrast, only a small subset of variables showed limited or no evidence of overall differences; in particular, the microbiological safety indicators aerobic mesophilic and total coliforms were among the weakest signals in the ANOVA analysis (**Figure 2a**), suggesting the absence of consistent separation among materials under Tukey’s test for these variables. While their detailed full mean ± Standard Deviation values ​​and Tukey groupings are available in **Supplementary Table 1**.

Building on this global inference, the normalized heatmap in **Figure 2b** (means with Tukey’s HSD groupings) provides an integrated view of the most extreme contrasts and their concordance with the multivariate clustering structure. Within the biofunctional domain, 13FLA is unequivocally positioned at the upper extreme of the antioxidant gradient, reaching the highest values for TPC (683.83), FRAP (3118.54), DPPH (1830.45), and ORAC_H (20248.62), and separating sharply from the remaining varieties, which largely occupy substantially lower ranges (e.g., TPC typically< ~200 in non-purple materials). This variety-level outlier pattern is recapitulated at the cluster level: Cluster 3 concentrates the strongest biofunctional profile (TPC = 683.83; FRAP = 3118.54; DPPH = 1830.45; ORAC_H = 20248.62), whereas the other clusters remain markedly lower (see **Supplementary Table 1** for complete values and Tukey groupings). Importantly, these results indicate that intra-specific variability is not only statistically significant but also technologically actionable, defining a collapsible group with exceptional antioxidant performance.

**Figure 3:**
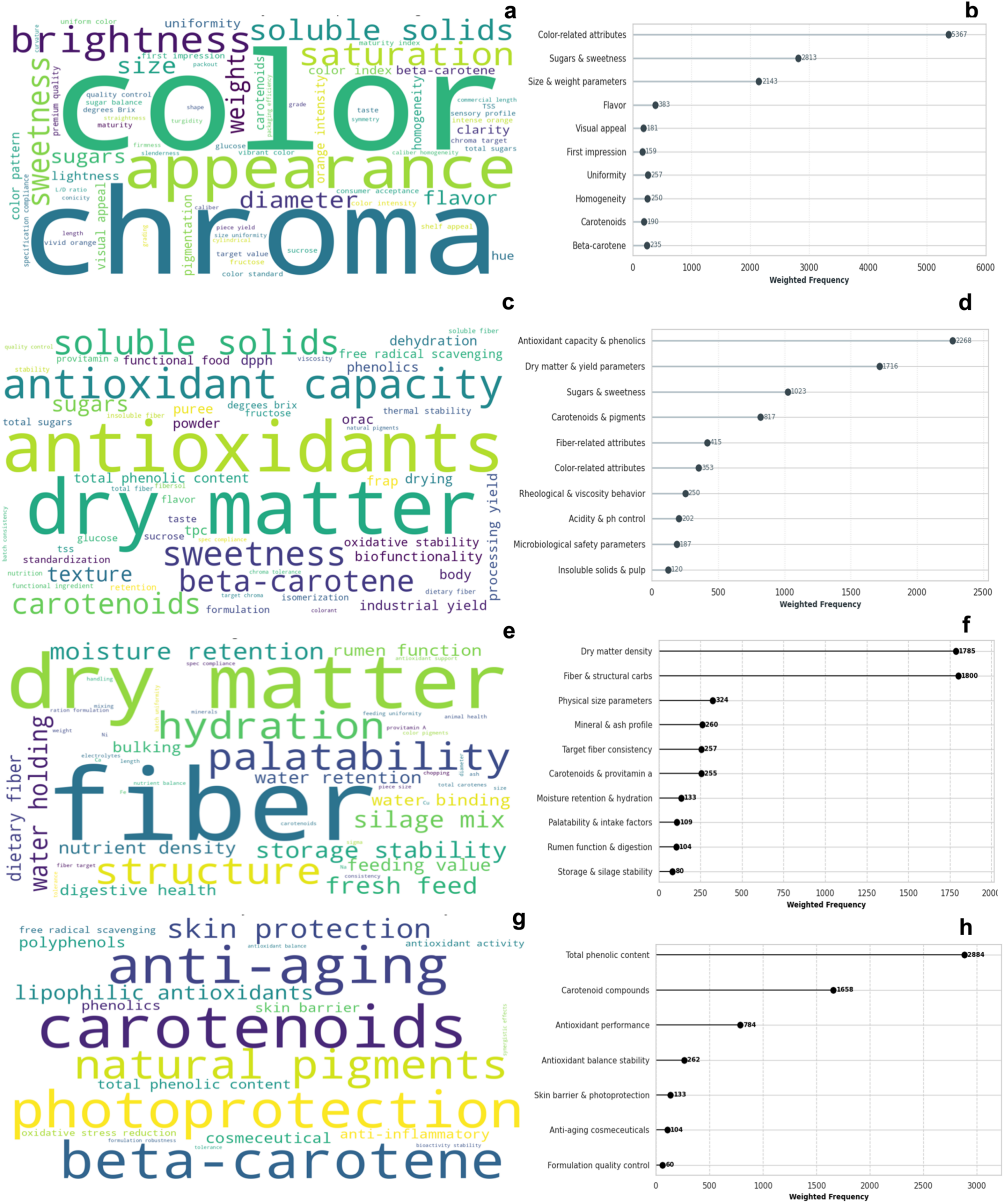
Word clouds **(a, c, e, g)** and lollipop plots **(b, d, f, h)** summarizing the key carrot quality attributes for fresh consumption, functional foods, pet feed, and natural cosmetics, respectively. The size of the terms in the word clouds and the length of the lines in the lollipop plots reflect their relative mathematical contribution and weight hierarchy within each industry-specific quality index.

Colorimetric traits exhibit similarly pronounced differentiation, consistent with the dominant ANOVA signals in **Figure 2a**. The pale materials 11NAN and 12NAN (assigned to the light-colored cluster) show the highest external lightness (L_C = 63.99 and 63.08, respectively) alongside a_C values near zero or negative (−1.93 in 11NAN; 0.82 in 12NAN), reflecting minimal red/orange contribution. At the opposite extreme, 13FLA displays a dramatic reduction in L_C (25.27) and an overall chromatic signature consistent with dark pigmentation, accompanied by an exceptionally low h_C (11.30). At the cluster scale, Cluster 2 maximizes lightness (L_C = 63.54) and high hue-angle values characteristic of pale roots (h_C = 94.04), whereas Cluster 3 defines the dark pole (L_C = 25.27; h_C = 11.30). Clusters 1 and 4 group intermediate, “orange-type” profiles with comparatively homogeneous lightness and hue (**Figure 3b**; **Supplementary Table 1**).

For compositional and processing-relevant attributes, the dataset also shows extremes with clear technological interpretation. In total soluble solids (Brix), the highest values occur in 6KUR (8.75) and 13FLA (8.52), while the pale materials 11NAN–12NAN are among the lowest (6.77–6.83). This gradient is mirrored by cluster means (Cluster 3 > Cluster 4 > Cluster 1 > Cluster 2). Similarly, dry matter peaks in 6KUR (11.85) and 13FLA (11.64), contrasting with 12NAN (8.94) and 11NAN (9.16), indicating that the pale cluster tends toward higher moisture and reduced solids, whereas orange and purple materials converge on more concentrated matrices. Accordingly, moisture reaches its maximum in 12NAN (91.06) and 11NAN (90.84) and its minimum in 6KUR (88.15), with a clear cluster-level separation (Cluster 2 > Cluster 1 > Cluster 3 ≈ Cluster 4; **Supplementary Table 1**).

Provitamin A–related traits are also strongly differentiated and aligned with chromatic profiles. Total carotenoids attain their highest levels in 6KUR (2.42), 5BER (2.12), and 1NAN (2.07), whereas the pale cluster shows near suppression (11NAN = 0.00; 12NAN = 0.15). A comparable pattern is observed for β-carotene, with maxima in 8NAN and 6KUR (0.40) and null values in 11NAN, 12NAN, and 13FLA (0.00). Thus, clusters reflect not merely external appearance but distinct compositional trajectories (**Figure 3b**; **Supplementary Table 1**). At the cluster level, Clusters 4 (deep orange) and 1 sustain the highest carotenoid/β-carotene load, while Clusters 2 (pale) and 3 (purple) represent the lower extremes.

Dietary fiber metrics further reinforce the distinctiveness of the biofunctional cluster. Total fiber is maximized in 13FLA (4.15), exceeding the central range of most materials (~2.3–3.3), and soluble fiber is also elevated in 9NAN (0.95) and 13FLA (0.89), contrasting with varieties showing null values (e.g., 7NAN and 14BER). At the cluster scale, Cluster 3 again represents the highest total fiber mean (4.15), indicating a dual biofunctional package (antioxidant capacity plus fiber) that is relevant for functional food formulation (**Figure 3b**; **Supplementary Table 1**).

Finally, physical traits show meaningful though comparatively less dominant variation that can influence market segmentation. Carrot weight spans from 98.56 (13FLA) to 200.82 (9NAN), while root length is highest in 2CHAN (22.55) and 12NAN (23.00). These gradients support differentiation between fresh-market typologies and processing-oriented materials and are visually integrated into the overall heatmap structure (**Figure 2b**).

### 3.3 Industry-tailored prioritization, usability modeling, and desirability benchmarking of carrot quality traits across promising end-use vocations of cultivated carrots in the Colombian Andean region

#### 3.3.1 Prioritization patterns for quality characteristics specific to promising industries

**Figure 3** integrates qualitative salience, represented by word clouds, with quantitative prioritization, represented by lollipop plots, to formalize four industry-specific quality frameworks. In the word clouds, term size reflects the relative semantic contribution of each attribute group to the corresponding quality logic, whereas in the lollipop plots, bar length denotes the weighted frequency of each consolidated attribute category.

For fresh consumption, **Figure 3a** shows a quality logic dominated by visual and sensory-market attributes, particularly chroma, appearance, brightness, saturation, sweetness, diameter, flavor, and size-related descriptors. This pattern is quantitatively reinforced in **Figure 3b**, where Color-related attributes represented the highest-weighted category (5367), followed by Sugars & sweetness (2813) and Size & weight parameters (2148). These three categories define the main consumer-facing quality axis for fresh-market carrot selection, combining visual attractiveness, perceived sweetness, and physical commercial standards. Lower-weighted but still relevant descriptors included Flavor (383), Uniformity (257), Homogeneity (250), Beta-carotene (235), Carotenoids (190), Visual appeal (181), and First impression (159), indicating that specific sensory and pigment-related traits act as complementary rather than dominant drivers once the main visual and size-related attributes are satisfied.

For functional foods and processing-oriented applications, **Figure 3c** shifted the vocabulary toward bioactivity, processing performance, and matrix concentration, with terms such as antioxidant capacity, antioxidants, dry matter, soluble solids, sweetness, beta-carotene, carotenoids, phenolics, texture, and processing yield. The quantitative hierarchy in **Figure 3d** confirmed this structure. Antioxidant capacity & phenolics was the dominant category (2268), followed by Dry matter & yield parameters (1716), Sugars & sweetness (1023), and Carotenoids & pigments (817). This indicates that functional-food suitability is driven by the joint contribution of bioactive potential, solids concentration, and pigment-associated nutritional value. Secondary contributors included Fiber-related attributes (415), Color-related attributes (353), Rheological & viscosity behavior (250), Acidity & pH control (202), Microbiological safety parameters (187), and Insoluble solids & pulp (120), reflecting the importance of formulation stability, processability, and safety as supporting criteria.

For pet feed, **Figure 3e** was predominantly characterized by concepts related to dry matter, fiber, hydration, palatability, water retention, nutrient density, structure, and storage stability. This semantic structure reflects the relevance of biomass concentration, structural carbohydrates, and formulation behavior in feed-oriented quality assessment. The quantitative prioritization in **Figure 3f** showed that Fiber & structural carbs (1800) and Dry matter density (1785) were the dominant components of the pet-feed index. These were followed by Physical size parameters (324), Mineral & ash profile (260), Target fiber consistency (257), and Carotenoids & provitamin A (255), indicating that nutritional density and structural functionality are complemented by particle uniformity, mineral composition, and provitamin A-related attributes. Lower-weighted descriptors included Moisture retention & hydration (133), Palatability & intake factors (109), Rumen function & digestion (104), and Storage & silage stability (80), which function mainly as operational and formulation-support variables.

For natural cosmetics, **Figure 3g** emphasized bioactivity and cosmetic functionality, with prominent terms associated with photoprotection, carotenoids, anti-aging, skin protection, beta-carotene, natural pigments, phenolics, lipophilic antioxidants, and polyphenols. The quantitative ranking in **Figure 3h** showed that Total phenolic content was the highest-weighted category (2884), followed by Carotenoid compounds (1658) and Antioxidant performance (784). This hierarchy indicates that cosmetic suitability is primarily driven by phenolic bioactivity, pigment-associated lipophilic compounds, and integrated antioxidant performance. Additional categories included Antioxidant balance stability (262), Skin barrier & photoprotection (133), Anti-aging cosmeceuticals (104), and Formulation quality control (60). Overall, the updated structure demonstrates coherence between the consolidated semantic categories, laboratory-derived compositional variables, and the mathematical logic of each industry-specific quality index.

#### 3.3.2 Construction of multi-criteria usability indices adapted to promising industries

Based on the industry-specific quality logics presented in section 3.3.1, this research translated these perceived priorities into a set of four usability indices that operationalize the carrot-like quality as industry-specific decision functions. Thus, the indices constitute a key outcome of this research: a mathematical framework that converts multidimensional quality-related variables into comparable and industry-calibrated suitability scores. This demonstrates that industries do not solely maximize the magnitude of individual variable traits but rather prioritize predictable performance around their target business specifications and value proposition to their customers.

For fresh consumption, the proposed index D Fresh (i) encodes the consumer-facing valuation system dominated by visual and sensory cues (**Figures 3a, b**), where market acceptance is strongly driven by perceived freshness, external appearance, and sweetness proxies. Accordingly, the index combines soluble solids (°Brix), an external color index derived from CIELAB parameters, and a size-uniformity component integrating weight, length, and diameter, which together represent sweetness expectation, visual appeal, and commercial grading. Importantly, a Gaussian penalty term is applied to the deviation of external chroma from a predefined target (C*_0), reflecting the retail reality that color uniformity and closeness to an expected reference often outweigh simply maximizing saturation. The resulting formulation is:


$$DFresh\left(i\right)=(0.30u\left(IC\right)+0.25u\left({}^{\circ}Brix\right)+0.20u\left(Size\right)+0.25\;exp\left(-\frac{{\left(Ci*-C*0\right)}^{2}}{\left(2\sigma^{2}\;C*\right)}\right)$$

Where: $IC=\frac{C^{*}.a^{*}}{L}$A functional food index (*DFunctional*) was formulated to identify carrot varieties suitable for industrial transformation into juices, purées, pulps, and natural ingredients or colorants. This index integrated variables associated with formulation performance and technological yield, including soluble solids or total sugars, carotenoid content, an internal color index, and dry matter percentage. These variables reflect both the sensory profile of the final product and its functional value and processing efficiency. A Gaussian penalty was applied to internal chroma (CI_II​) deviations to represent the need for chromatic standardization in agroindustrial processes, where consistency between batches is more critical than maximizing pigment intensity. Accordingly, the index favors varieties with stable internal color profiles that minimize formulation adjustments and reprocessing requirements. The index is defined as:


$$DFunctional\left(i\right)=0.18u\left({}^{\circ}Brix\right)+0.22u\left(DryMatter\right)+0.22u\left(Antiox\right)+0.16u\left(\beta Carotene\right)+0.12u\left(Fibersol\right)\ldots +0.10\;exp\left(-\frac{{\left(Ci*-C*0\right)}^{2}}{\left(2\sigma^{2}\;C*\right)}\right).$$

The animal feed index was specifically adjusted for wet pet food applications, where sensory acceptance, energy contribution, and digestive functionality are particularly relevant. Dry matter content was incorporated as a key indicator of technological yield and product stability, while total dietary fiber was included due to its importance for gastrointestinal health and regulation of intestinal transit. Root size was considered as a variable associated with ingredient manageability and acceptance in wet formulations, where visual perception and texture influence intake. Essential mineral content was integrated to reflect overall nutritional value, and total carotenoids were included due to their documented functional and antioxidant roles in companion animal diets. A Gaussian penalty function based on deviations of fiber content from an optimal reference value was applied to favor balanced fiber profiles and penalize excesses or deficiencies that could negatively affect palatability and digestibility. The index is defined as:


$$DPetfeed\left(i\right)=0.30u\left(DryMatter\right)+0.30u\left(Fiber_{sol}\right)+0.10u\left(Size\right)+0.10u\left(Minerals\right)+0.10u\left(TotalCarotenes\right)\ldots +0.10\;exp\left(-\frac{{\left(Fi*-F*0\right)}^{2}}{\left(2\sigma^{2}\;Fiber\right)}\right)$$

Finally, a natural cosmetics index was designed to identify carrot varieties with potential for the development of plant-based cosmetic ingredients, prioritizing bioactive compound content and functional stability. This index integrated total carotenoids, total phenolic compounds (TPC), and a composite antioxidant index derived from FRAP, DPPH, and ORAC assays. Unlike other sectors, the Gaussian penalty was applied to deviations in the antioxidant profile, calculated as the dispersion among normalized values obtained from the different analytical methods. This penalization strategy favors varieties with consistent and reproducible antioxidant behavior, a key requirement for the standardization of cosmetic extracts and active ingredients, where functional consistency across batches is essential for formulation and commercialization. The index is defined as:


$$DNatural\;C\left(i\right)=0.40u\left(TPC\right)+0.30u\left(TotalCarotenes\right)+0.20u\left(Antiox\right)+0.10\;exp\left(-\frac{yi^{2}}{\left(2\sigma^{2}Antiox\right)}\right)$$

Where:


Antiox=0.35(ORAC)+0.25(FRAP)+0.25(DPPH)+0.15(TPC))

$$yi=\sqrt{\frac{{\left(uORAC-\underline{u}\right)}^{2}+{\left(uFRAP-\underline{u}\right)}^{2}+{\left(uDPPH-\underline{u}\right)}^{2}+{\left(uTPC-\underline{u}\right)}^{2}}{4}}$$

**Figure 4:**
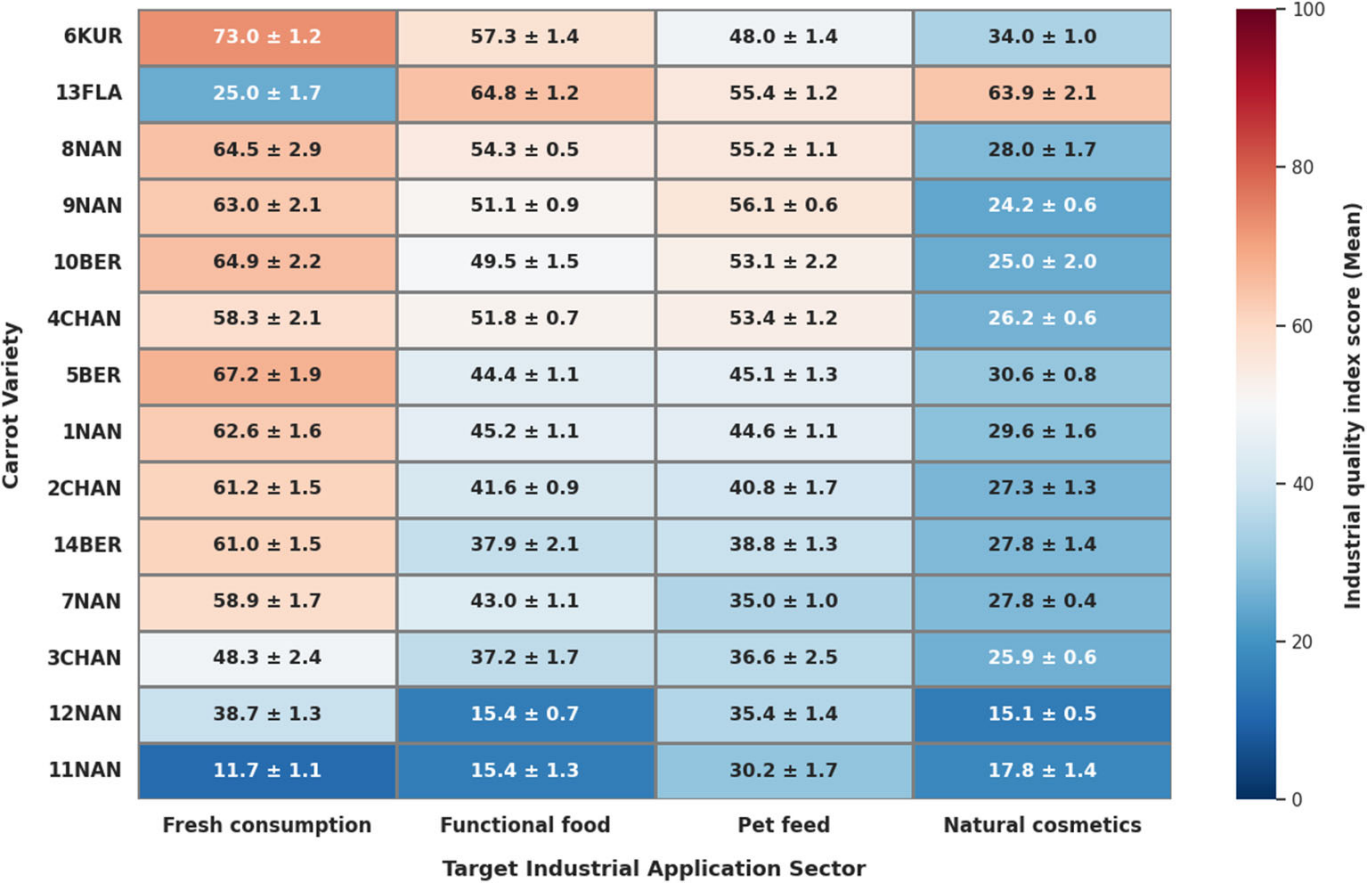
Heatmap of industry-specific usability indices for carrot varieties. Mean usability index values (0–100 scale) and their corresponding standard errors (± SE) are displayed within each cell for fresh consumption, functional foods, pet feed, and natural cosmetics, calculated using a multicriteria decision-making framework. Carrot varieties were hierarchically ordered along the vertical axis according to their overall mean performance across all industrial sectors to highlight multi-industry versatility. The divergent color scale represents lower (blue) to higher (red) levels of industrial suitability and raw material valorization potential.

Using the four industry-tailored usability indices (DFresh, DFunctional, DPetfeed, and DNaturalC) derived from the multicriteria utility functions defined in this study, standardized suitability scores (0–100) were computed for each carrot variety and their cross-sector performance was summarized in **Figure 4**. Heatmap of industry-specific usability indices for carrot varieties. Mean usability index values are shown for fresh consumption, functional foods, pet feed, and natural cosmetics, and varieties are ordered by their overall mean performance. This matrix provides a decision-oriented synthesis of how multidimensional quality attributes translate into differentiated industrial suitability.

The **Figure 4** reveals a marked trade-off between fresh-market aptitude and performance in higher value-added industrial applications, indicating that no single genotype maximizes all end-use criteria simultaneously. For fresh consumption, the highest index scores were observed for 6KUR (73.0 ± 1.2), followed by 5BER (67.2 ± 1.9), 10BER (64.9 ± 2.2), 8NAN (64.5 ± 2.9), and 9NAN (63.0 ± 2.1), consistent with favorable combinations of soluble solids, external color-related performance, and commercial-scale morphological uniformity. In contrast, the lowest fresh-market suitability was recorded for 11NAN (11.7 ± 1.1) and 12NAN (38.7 ± 1.3), reflecting profiles that are comparatively less aligned with consumer-driven visual and sensory thresholds; these lower fresh-market performers tended to show relatively greater alignment with processing-oriented indices, reinforcing functional differentiation between “appearance-driven” and “technology-driven” quality dimensions. For functional foods, a specialization pattern emerged, with 13FLA exhibiting the highest suitability (64.8 ± 1.2), followed by 6KUR (57.3 ± 1.4) and 8NAN (54.3 ± 0.5), and then a second tier including 4CHAN (51.8 ± 0.7) and 9NAN (51.1 ± 0.9). This distribution supports the interpretation that varieties scoring highly in this category combine attributes that enhance transformation performance and product value (e.g., higher solids density, bioactive-related contributions, and overall technological robustness), rather than optimizing fresh-market aesthetics. At the opposite extreme, 11NAN (15.4 ± 1.3) and 12NAN (15.4 ± 0.7) showed limited alignment with the functional-food index, indicating restricted suitability for ingredient-oriented processing under the weighting scheme defined for this vocation. The pet feed index displayed a comparatively narrower spread, consistent with several varieties meeting baseline nutritional and technological requirements, yet distinct leaders were still evident. The highest scores were achieved by 9NAN (56.1 ± 0.6), 13FLA (55.4 ± 1.2), 8NAN (55.2 ± 1.1), and 4CHAN (53.4 ± 1.2), closely followed by 10BER (53.1 ± 2.2). These varieties likely benefit from balanced contributions of dry matter, fiber-related functionality, mineral composition, and total carotenoids, aligning with formulation priorities in wet pet food (digestive functionality, matrix handling, and stability). Lower pet-feed suitability was observed for 11NAN (30.2 ± 1.7) and 7NAN (35.0 ± 1.0) and 12NAN (35.4 ± 1.4), indicating a less favorable balance relative to the defined optimal targets. For natural cosmetics, the suitability landscape appeared more restrictive, with 13FLA (63.9 ± 2.1) as the top performer, followed by 6KUR (34.0 ± 1.0) and a mid-range group including 5BER (30.6 ± 0.8) and 1NAN (29.6 ± 1.6). The lowest cosmetics suitability was observed for 12NAN (15.1 ± 0.5) and 11NAN (17.8 ± 1.4), suggesting limited potential for standardized cosmeceutical ingredient development under the antioxidant- and pigment-centered logic of DNaturalC. Notably, the cosmetics index embeds a Gaussian penalization linked to the consistency of antioxidant behavior across assays, thereby distinguishing varieties that combine high carotenoid/phenolic potential with reproducible functional performance, a key requirement for batch-to-batch standardization of cosmetic extracts and active ingredients.

#### 3.3.3 Desirability spaces based on usability and performance benchmarks for carrots in promising industries

**Figure 5:**
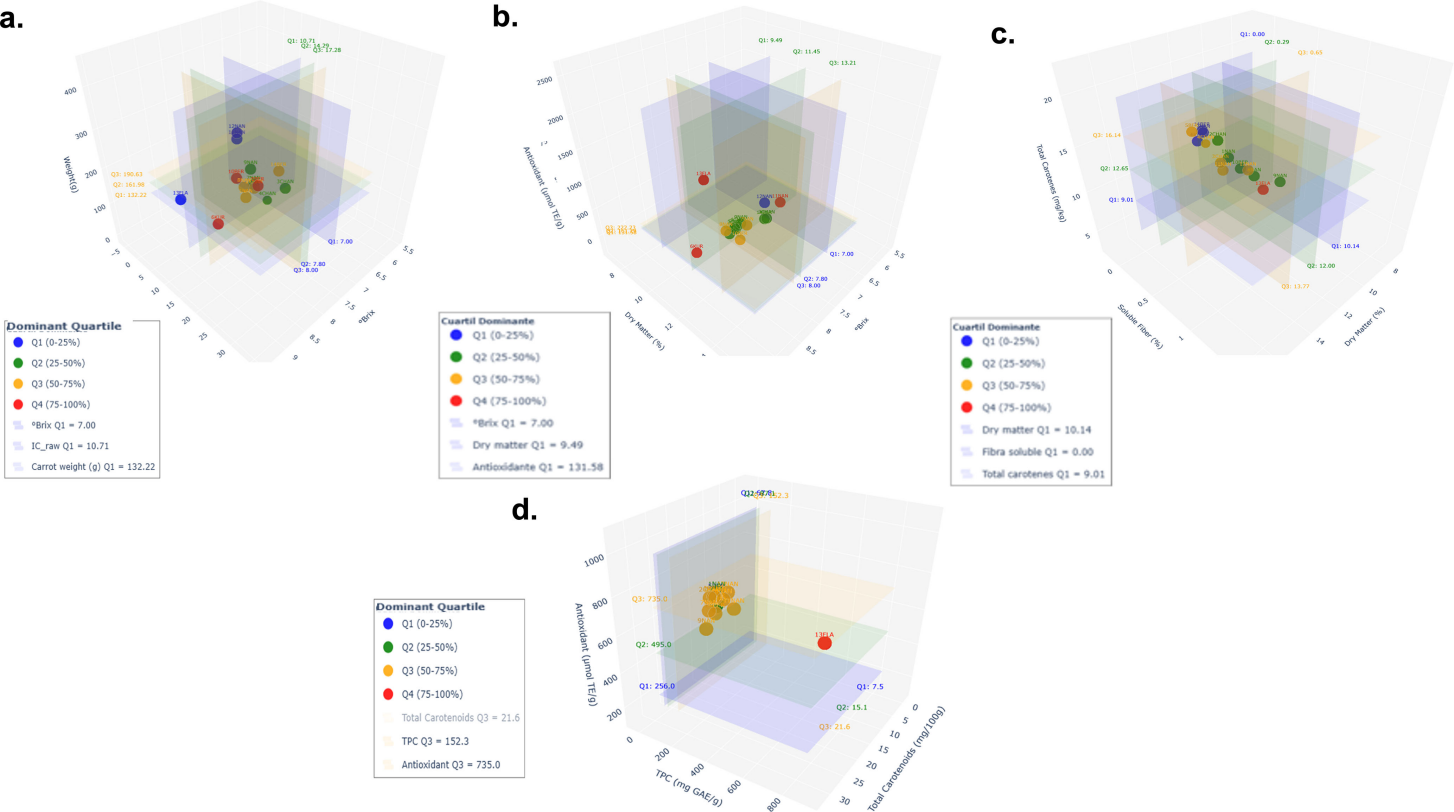
Three-dimensional, quartile-based spaces (Q1–Q4) were constructed using the most relevant variables included in the industry-specific quality indices: **(a)** fresh consumption (°Brix, color index and carrot weight); **(b)** functional foods (°Brix, dry matter and antioxidant index); **(c)** pet feed (dry matter, soluble fiber and total carotenoids); and **(d)** natural cosmetics (total carotenoids, total phenolic content and antioxidant activity).

**Figure 5** synthesizes the competitive positioning of carrot accessions within industry-specific, three-dimensional desirability spaces for (a) fresh consumption, (b) functional foods, (c) pet feed, and (d) natural cosmetics, using the key variables embedded in each quality index (°Brix, IC, root weight; °Brix–dry matter–antioxidant index; dry matter–soluble fiber–total carotenoids; total carotenoids, TPC, antioxidant activity, respectively). In each panel, variety centroids (mean values) are projected onto orthogonal planes defining the quartile thresholds (Q1–Q4), thereby converting multivariate dispersion into interpretable decision ranges. This quartile partitioning enables the identification of varieties that consistently occupy the upper-performance volume (Q3–Q4) in the most influential traits, and thus supports the proposal of operational selection ranges aligned with end-use requirements.

For fresh consumption (**Figure 5a**), desirability is maximized when sweetness proxies, external visual performance, and commercial caliber converge within the upper quartiles. Soluble solids spanned approximately 6.0–9.0°Brix, with a relatively narrow interquartile spread, supporting the use of ≥ Q3 (≈8.0°Brix) as a pragmatic threshold for higher perceived sweetness. The external color index (IC) showed markedly larger dispersion (approximately −0.94 to 31.08), confirming its role as a primary discriminator of market-facing quality; thus, IC ≥ Q3 (17.3) can be interpreted as a minimum benchmark for vivid, visually appealing external coloration. Root weight exhibited the highest variability (approximately 45–395 g), and the mid–upper quartile corridor (Q2–Q3; 162–191 g) represents a commercially desirable caliber range that balances consumer preference and packaging/standardization constraints. Varieties located in the upper multivariate volume (simultaneously ≥Q2–Q3 across °Brix, IC, and weight) represent the most robust candidates for fresh markets, whereas accessions that excel in one axis but fall below quartile thresholds in the others illustrate that fresh-market suitability is intrinsically multicriteria rather than driven by a single trait.

For functional foods (**Figure 5b**), the desirability space is governed by the joint optimization of bioactivity and processing feasibility, formalized here by the antioxidant index, dry matter, and °Brix. The antioxidant index displayed strong right-skewness and a wide dynamic range, indicating substantial inter-varietal heterogeneity; quartile thresholds define practically useful strata, with Q3 (≈143) and above delimiting high-bioactivity materials. Dry matter values clustered around 11–13%, suggesting that the most actionable differentiation in this vocation is driven by the antioxidant axis, provided dry matter remains within the technologically acceptable band. A large subset of varieties occupies intermediate antioxidant performance (approximately Q2), consistent with broadly suitable materials for routine functional products. In contrast, the varieties positioned in Q4 for antioxidant performance (notably the accessions plotted at the extreme high end, such as 13FLA) reflect a distinct nutraceutical-grade profile, where antioxidant capacity becomes the dominant driver of desirability and can justify specialization even if sweetness proxies remain moderate.

For pet feed (**Figure 5c**), the desirability space emphasizes nutritional functionality and formulation stability rather than extremes of bioactivity. Dry matter again concentrates within a narrow window consistent with wet-formulation requirements, while total carotenoids define an added-value axis, with ≥Q3 (≈13 mg/kg) serving as a useful benchmark for enhanced functional/pigment contribution. Soluble fiber exhibited generally low values (often approaching Q1 ≈ 0), indicating that selection is driven less by maximizing fiber and more by achieving a balanced fiber profile compatible with palatability and digestibility constraints. Consequently, the most desirable materials tend to occupy Q2–Q3 across dry matter, soluble fiber, and carotenoids, yielding a profile optimized for ingredient consistency, gastrointestinal functionality, and stable product performance. The comparatively compressed quartile dispersion across varieties supports the interpretation of pet feed as an intermediate valorization pathway, where many accessions meet baseline requirements, but only a subset achieves a well-balanced upper-quartile configuration.

For natural cosmetics (**Figure 5d**), the desirability space was highly restrictive, requiring concentrated and coherent bioactive profiles suitable for extract standardization. Total carotenoids exhibited well-defined quartile thresholds (Q1: 7.5, Q2: 15.1, and Q3: 21.6 mg/100g), while TPC established intermediate decision boundaries, with a Q3 threshold of 152.3 mg GAE/g. Antioxidant activity generated strong discrimination limits (Q1: 256, Q2: 495, and Q3: 735 μmol TE/g), enabling robust differentiation of accessions according to their redox-related performance. The three-dimensional spatial distribution revealed that most accessions clustered within an intermediate-to-high performance region characterized by elevated antioxidant activity and substantial phenolic accumulation, although generally limited by moderate carotenoid concentrations. However, premium cosmetic applications require the simultaneous maximization and balance of all three bioactive dimensions. Under this framework, 13FLA clearly emerged as an isolated Q4 candidate. Its distinct positioning within the multivariate space reflected not only a high total carotenoid concentration, but also superior compositional balance across antioxidant and phenolic dimensions, making it the only genotype capable of consistently satisfying the stringent reproducibility requirements associated with high-end cosmeceutical ingredient development. Conversely, the dense aggregation of accessions within the Q3 region demonstrated that although elevated antioxidant performance is relatively common within the evaluated germplasm, achieving the integrated multivariate balance observed in 13FLA remains uncommon. This pattern reinforces the importance of the proposed 3D spatial framework for identifying truly standardized raw materials for premium cosmetic applications.

Based on these findings, and to establish clear operational guidelines for the agro-industrial sector, the identified Q3 thresholds were integrated into a unified predictive decision-making matrix (**Table 1**). This framework transforms the descriptive quartile partitioning of the evaluated germplasm into standardized baseline criteria for industrial suitability, enabling the rapid screening and prioritization of new candidate varieties.

**Table 1:** Unified matrix of industrial suitability selection thresholds.

Target industrial sector	Key quality marker	Minimum advised quality standard (Q3​ threshold)	Predictive suitability decision rule (For testing varieties)
Fresh Consumption	Brix Color Index*(CI)* Carrot Weight	≥ 7.88 ≥ 12.46 ≥ 150.22	Highly Recommended: A testing variety is classified as Premium Fresh Market only if it achieves or exceeds all three metrics simultaneously
Functional Foods	Brix Dry Matter (%) Antioxidant Index	≥ 7.88 ≥ 11.45% ≥ 481.28	Functional Food Fit: Meets the minimum matrix concentration required for efficient industrial bioactive extraction, concentration, and enrichment.
Pet Feed	Dry Matter (%) Soluble Fiber (%) Total Carotenoids	≥ 13.77% ≥ 0.45% ≥ 14.14 mg/100g	Feed Optimization Grade: Guarantees nutritional fiber quality and baseline natural pigmentation yields for premium animal feed manufacturing
Natural Cosmetics	Total Carotenoids TPC (Phenols) Antioxidant Activity	≥ 21.60 mg/100g ≥ 152.30 mgGAE/g ≥ 735.00 μmol TE/g	Cosmeceutical Grade (Maximum Restriction): Exceptional tier. Only varieties entering this space (e.g., 13FLA) guarantee the high redox stability needed for premium formulations.

#### 3.3.4 Quality and safety analysis of carrots and regulatory criteria at the industry level

Ten health and safety variables were analyzed in 14 carrot varieties and grouped into four clusters: 1, 2, 3, and 4. The clusters showed significant differences in mesophilic aerobic microorganism counts (p< 0.05). Cluster 3 presented the highest values (172,850 ± 281,487 CFU/g), compared to Cluster 4, which reported the lowest values (35,693 ± 33,804 CFU/g). Clusters 1 and 2 showed intermediate values with no significant differences between them. A similar pattern emerged for total coliforms. Cluster 1 presented the highest average value (59,887 ± 169,802 CFU/g);, compared to cluster 4 which reported the lowest values (10,209 ± 15,779 CFU/g);, while Clusters 2 and 3 reported intermediate values and no clear significant differences with respect to Cluster 1 or 4. For molds and yeasts, values of<10 CFU/g were reported, with no significant differences between clusters. For the qualitative variables of absence/presence, applied to the case of *Salmonella* spp. it was reported as absence/25 g and pesticides as not detectable in all samples.

Ensuring the quality and safety of carrots (*Daucus carota* L.*)* in their various industrial uses is based on a preventive and risk-based approach, aligned with current international and national regulatory frameworks. At the international level, the Codex Alimentarius Commission (Food and Agriculture Organization of the United Nations and World Health Organization, 2023) promotes the application of Good Agricultural and Hygiene Practices, the use of microbiological indicators such as *Escherichia coli*, and compliance with maximum residue limits for pesticides as the basis of international trade. These principles are reinforced through food safety management systems based on HACCP integrated into the ISO 22000 standard. In high-value markets, such as the European Union and the United States, these guidelines are operationalized through preventive controls on fresh produce, mandatory microbiological criteria for ready-to-eat foods, verification of MRLs considering processing factors, and evaluation of chemical contaminants in animal feed and cosmetics (European Parliament & Council, 2005; European Commission, 2005b; European Parliament & Council, 2009; U.S. Food and Drug Administration, 2015). In Colombia, this framework is implemented through the Good Agricultural Practices established by the Resolución 30021 de 2017, 2017, the inspection and surveillance carried out by INVIMA in accordance with Law 1122 of 2007, and the regulation of cosmetic products under Decision 833 of 2018 of the Andean Community (Instituto Nacional de Vigilancia de Medicamentos y Alimentos - INVIMA.), complemented by the application of Colombian Technical Standards harmonized with the Codex (**Supplementary Table 2**), which supports the need for continuous, risk-based, market-oriented food safety monitoring programs to guarantee consumer protection and product competitiveness.

### 3.4 Operational workflow for decision-support for agrobiodiversity based raw-material allocation.

#### 3.4.1 Operational workflow for decision-support and transferable bioprospecting

**Figure 6:**
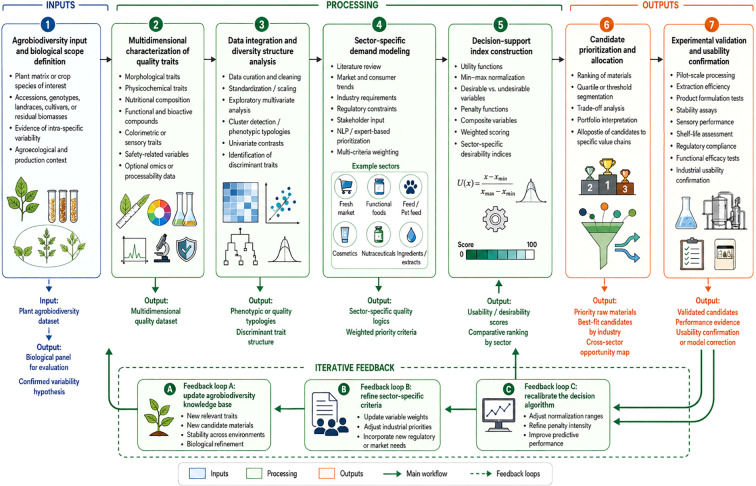
Generalized decision-support workflow for agrobiodiversity-based bioprospecting and raw-material allocation in plant matrices.

To operationalize the proposed desirability-index framework, the analytical sequence was synthesized into a generalized decision-support workflow for agrobiodiversity-based raw-material allocation (**Figure 6**). The workflow shows how a research or industrial team can use the framework to move from a biologically diverse plant panel to a prioritized set of candidate materials for specific value chains. Although the present study applied the approach to carrot, the workflow was designed to be transferable to other plant matrices, provided that intra-specific variability is first confirmed.

The workflow begins with the definition of an agrobiodiversity input, such as accessions, genotypes, cultivars, landraces, or residual biomasses from a target species. These materials are then subjected to multidimensional characterization, integrating quality-related variables such as morphological, physicochemical, nutritional, colorimetric, biofunctional, sensory, processability, and safety-related traits. This information constitutes the primary quality dataset used to describe the biological and technological variability of the plant matrix. In the next stage, the dataset is standardized and analyzed to identify phenotypic or quality typologies, discriminant traits, and statistically relevant contrasts among materials. These biological outputs are then connected with sector-specific demand modeling, where literature evidence, market trends, regulatory requirements, stakeholder input, and multi-criteria weighting are used to define the quality logic of each target industry. The core decision step is the construction of sector-specific usability indices. In this phase, desirable and undesirable variables are normalized, weighted, and integrated through utility and penalty functions to generate comparative desirability scores. These scores allow the research team to rank materials by industry, detect cross-sector trade-offs, and select the most promising candidates for downstream validation. The final stage corresponds to experimental validation and usability confirmation. Prioritized materials should be tested through pilot-scale processing, extraction efficiency, formulation assays, stability studies, sensory or functional performance tests, shelf-life evaluation, regulatory compliance, and industrial usability trials. Validation results are then fed back into the workflow to update the agrobiodiversity knowledge base, refine sector-specific criteria, and recalibrate the decision algorithm. Therefore, the proposed workflow is not a linear classification system, but an adaptive decision-support model for continuous improvement of plant-based bioprospecting and raw-material allocation.

## 4 Discussion

Carrot valorization has traditionally been constrained by a reductionist quality paradigm in which physicochemical, compositional, functional, and technological traits are interpreted as isolated endpoints rather than as integrated phenotypic configurations that determine industrial performance. Although the biochemical richness of colored carrot germplasm, including carotenoids, phenolics, and antioxidant capacity, has been widely documented, these attributes are frequently analyzed independently, limiting their translation into decision-ready criteria for differentiated end uses (Pérez et al., 2023). This is particularly relevant because most industrial applications, including natural colorants, nutraceuticals, cosmetic actives, functional foods, and animal feed ingredients, depend on coordinated trait packages rather than on the maximization of a single marker. Pigment chemistry, compositional density, extractability, stability, safety, sensory acceptability, and bioactivity must therefore be evaluated jointly to ensure process robustness and product standardization (Riaz et al., 2022). Accordingly, stable carrot-derived colorants depend not only on pigment concentration but also on matrix composition and processing behavior, whereas nutraceutical positioning depends on multicomponent synergies and bioavailability rather than on a single antioxidant endpoint (Pérez et al., 2023; Soleti et al., 2020).

A central contribution of this study is the translation of sector-specific quality requirements into operational decision functions capable of ranking carrot materials across multiple value chains. The gap addressed is primarily pragmatic: industrial stakeholders, particularly in emerging or fragmented agro-industrial contexts, recognize quality as multidimensional but often lack clear criteria to determine which variables should be prioritized for each sector and which materials best satisfy those requirements under realistic sourcing conditions. The proposed desirability functions and multicriteria indices (DFresh, DFunctional, DPetfeed, and DNaturalC; Equations 1–5) formalize this translation by integrating trait utilities, sector-derived weights, and penalty terms for attributes that may compromise technological performance, homogeneity, or market suitability. Compared with univariate screening or informal expert appraisal, this index-based framework provides traceable and reproducible rankings that can support procurement, portfolio design, and early-stage R&D prioritization before undertaking full end-product validation (Cobb et al., 2019).

Beyond ranking individual genotypes, the results support a broader inference: carrot production in the Colombian Andean region can be approached as a differentiated bioeconomy platform if agrobiodiversity is managed as a strategic resource. The intra-specific variability observed under uniform agronomic conditions indicates that the evaluated materials express distinct trait packages aligned with different value niches, including pigment-rich matrices, antioxidant-dense ingredients, fiber-oriented feed components, and neutral-color substrates. This distinction is critical because differentiated industries generally demand functional performance, such as stability, extractability, formulation compatibility, and standardization, rather than isolated high values of a single analytical marker. Therefore, the most appropriate use of a given genotype should not be inferred from color or commercial type alone, but from its complete multidimensional profile and its alignment with sectoral requirements (Pérez et al., 2023; Soleti et al., 2020).

In this context, the integration of physicochemical, nutritional, colorimetric, safety-related, and biofunctional variables into a unified multivariate framework enabled the condensation of diversity into four internally coherent phenotypic groups. This structure should be interpreted as a functional typology rather than as a taxonomic, genetic, or purely visual classification. Although root color emerged as a highly informative phenotypic signal, particularly because pigment-linked optical traits and carotenoid-related variables contributed strongly to discrimination, the clusters were obtained from the complete standardized dataset. Thus, color acted as one component of broader industrially relevant trait syndromes. This interpretation is consistent with previous studies showing that multidimensional diversity in carrot germplasm can manifest as discrete, collapsible classes depending on trait scope, sampling depth, and the variables included in the analysis (Singh et al., 2022; Everitt et al., 2011). However, unlike studies focused primarily on population structure or provenance, the present framework emphasizes industrially exploitable phenotypic packages that can be operationalized as vocation-oriented classes (Valerga et al., 2023).

The separation of the purple material 13FLA illustrates the value of this approach. Its positioning in the multivariate space was not simply a consequence of external pigmentation, but reflected a coordinated functional profile associated with phenolic content and complementary antioxidant endpoints, including TPC, FRAP, DPPH, and ORAC-H. This pattern supports the interpretation that antioxidant capacity, captured through different analytical mechanisms, represents an integrated functional behavior linked to elevated phenolic biosynthesis and redox potential in dark-colored carrot materials (Pérez et al., 2023). In contrast, the pale materials were differentiated by reduced pigment-associated attributes and a distinct compositional profile, while the orange materials exhibited subtler but industrially meaningful variation in pigment density, soluble solids, dry matter, morphometric traits, and moisture balance. These gradients are relevant because the water–solids trade-off directly affects dehydration efficiency, extract concentration, shelf-life behavior, formulation yield, and processing costs (Sharma et al., 2012).

The industrial implication is that carrot quality cannot be reduced to a universal hierarchy of “best” and “worst” materials. Instead, quality is vocation-dependent. A genotype with strong antioxidant and phenolic expression may be highly suitable for functional ingredients or cosmetic applications but may not necessarily be optimal for fresh-market uniformity or dehydration yield. Conversely, orange materials with strong pigment density, adequate soluble solids, and favorable morphometric features may be better aligned with processing routes requiring standardized size, predictable yield, and carotenoid-rich matrices. Pale materials, although less suitable for carotenoid-based value propositions, may offer opportunities in neutral-color formulations, minimally processed matrices, or applications where chromatic neutrality is technologically desirable. This trade-off perspective is central to the proposed framework: the objective is not to identify a single superior genotype, but to allocate each material to the value chain in which its intrinsic constraints and advantages generate the greatest functional and economic coherence (Riaz et al., 2022; Cobb et al., 2019).

This portfolio logic is especially relevant for agrobiodiversity-based bioeconomy strategies. By structuring diversity into typologies, identifying actionable trait drivers, and translating sectoral priorities into usability scores, the cluster–index–valorization workflow provides a scalable template for bioprospecting in horticultural matrices. The framework can reduce early-stage screening costs by prioritizing candidates before product-scale validation and can improve resource efficiency by directing each raw material toward the application where it is most likely to generate differentiated value. In Andean production contexts, where multiple industries may compete for the same raw materials but often lack objective tools to justify price differentials, targeted contracts, or differentiated processing routes, this type of decision framework can contribute to more transparent and technically grounded value-chain engineering (Cobb et al., 2019; Riaz et al., 2022).

The results also reinforce the need to distinguish between traits that drive industrial differentiation and traits that mainly reflect production or handling conditions. Safety-related variables, including microbiological indicators, pesticide residues, and metals, are indispensable for determining suitability for regulated markets; however, in this study they showed limited discriminatory value among varieties. This suggests that, under the controlled production and handling conditions evaluated here, safety profiles were more strongly associated with lot-level management than with genotype-specific differentiation. Such behavior is consistent with preventive food-safety frameworks for fruits and vegetables, where risk management depends primarily on Good Agricultural Practices, Good Manufacturing Practices, HACCP-based systems, irrigation water quality, soil conditions, organic amendments, harvest hygiene, storage, and traceability rather than cultivar identity (Food and Agriculture Organization of the United Nations and World Health Organization,, 2023; Alegbeleye et al., 2018; Olaimat & Holley, 2012). Similarly, metal accumulation in root crops is strongly modulated by soil bioavailability and site-specific exposure, and varietal effects may become secondary or undetectable when exposure levels are low or relatively homogeneous (Ding et al., 2013; Novák et al., 2023). Therefore, the absence of strong varietal differentiation for these indicators should not be interpreted as reduced relevance of safety parameters, but rather as evidence that safety must be managed at the batch, production-system, and traceability levels. This is particularly important for high-value or highly regulated applications such as infant foods, functional ingredients, and cosmetic raw materials.

Several limitations should be considered when interpreting and extrapolating the proposed typologies and indices. First, cluster stability across environments, seasons, and production systems remains to be validated. Even when correlations among bioactive variables are maintained, absolute concentrations, extractability, and processing behavior can vary substantially as a consequence of genotype × environment interactions, postharvest conditions, and agronomic management, potentially reshaping multivariate distances and cluster membership (Pérez et al., 2023; Valerga et al., 2023). Second, although the four-cluster solution is statistically defensible and industrially interpretable, it remains a simplification of a more complex phenotypic continuum. Finer substructure, particularly within orange-rooted materials, may be relevant for specialized niches and could be explored through hierarchical sub-clustering or vocation-guided partitioning (Murtagh and Contreras, 2012). Third, the indices should be interpreted as early-stage decision-support tools rather than as substitutes for product-scale validation. Industrial adoption will require confirmatory trials assessing extraction yield, pigment stability, sensory performance, formulation compatibility, processing behavior, shelf-life, regulatory compliance, and downstream efficacy under real manufacturing conditions.

Future work should therefore prioritize multi-location and multi-season validation to quantify the stability of trait syndromes and explicitly model genotype × environment effects (Crossa et al., 2015). The integration of higher-resolution phenotyping, including untargeted metabolomics and rapid optical sensing platforms such as Vis–NIR or FT–NIR, could further improve the scalability of vocation-oriented selection (He et al., 2025). In parallel, genomic approaches would help clarify whether the observed phenotypic typologies are driven by functional differentiation, breeding history, population structure, or neutral genetic background, enabling marker-assisted deployment of materials with defined industrial vocations (Ellison et al., 2018; Meuwissen et al., 2001). Finally, end-product performance trials will be necessary to validate whether the proposed desirability indices predict technological behavior and market-relevant functionality in real product matrices.

Overall, this study reframes carrot agrobiodiversity as a structured reservoir of vocation-ready phenotypes rather than as unmanaged biological variability. Beyond the identification of differentiated materials, the main contribution is the development of a reproducible decision-support framework that connects multidimensional characterization with industry-oriented raw-material allocation. The workflow proposed in **Figure 6** extends this logic beyond carrot and can be extrapolated to agricultural production systems or plant matrices in which broad intra-specific variability and sufficient phenotypic distance among individuals, accessions, cultivars, or residual biomasses can be demonstrated. Under this premise, the quality of the model inputs depends on capturing the widest possible biological and phenotypic diversity, while its outputs must remain specific to industries with defined technical requirements and existing or emerging market opportunities for the target matrix. By combining multivariate typologies, trait interpretation, sector-specific desirability indices, and iterative validation, the proposed approach supports a transition from commodity-based sourcing toward adaptive portfolio-based valorization, where each material can be directed to the application in which its quality profile generates the greatest technical and economic value.

## 5 Conclusion

This study reframes carrot agrobiodiversity as a strategic bioeconomy asset by demonstrating that intra-specific variation is not “noise” to be standardized in raw materials, but rather a structured reservoir of trait combinations with tangible potential for industrial differentiation. Using an integrative framework combining multidimensional quality characterization, multivariate modelling, and univariate contrasts, we showed that the assessed materials can be consolidated into consistent phenotypic typologies that capture technologically meaningful gradients across multiple value chains. Building on this foundation, the main conceptual and applied contribution of the work is the explicit translation of sector-specific “quality logics” into reproducible decision functions (desirability-based indices) that enable transparent ranking and vocation-oriented allocation of raw materials, without relying on univariate screening or expert judgements that are difficult to trace and reproduce.

## Data Availability

The raw data supporting the conclusions of this article will be made available by the authors, without undue reservation.
