# Supplementary material for: A desirability index framework for the bioprospecting of carrots grown in the Andean region for fresh, feed, cosmetic and nutraceutical applications
Source: Front Plant Sci. 2026 Jun 5;17:1794157. doi: 10.3389/fpls.2026.1794157 (PMC13280928; doi:10.3389/fpls.2026.1794157)
Supplement: Supplementary file 1 [file DataSheet1.pdf]

Supplementary Material

1 Supplementary Figures and Tables

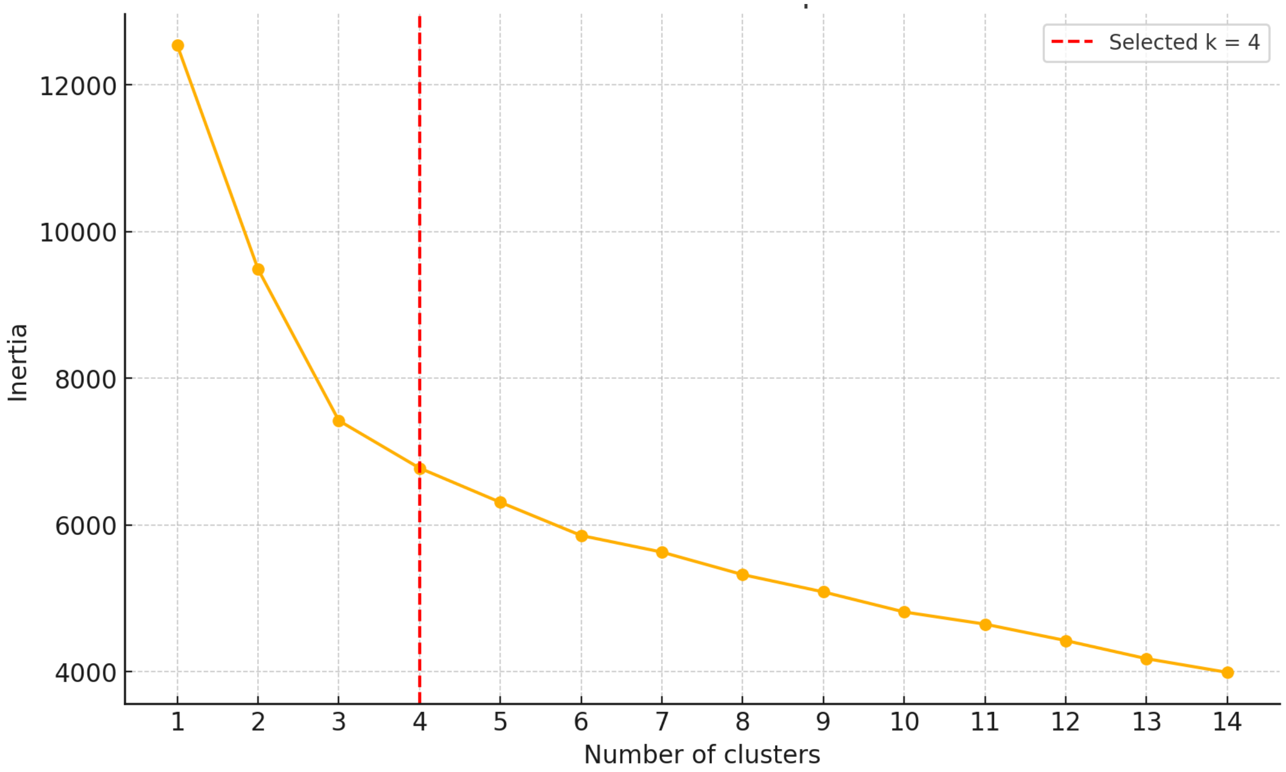

Supplementary Figure 1. Elbow method for selecting the optimal number of clusters (k) in K-means clustering of carrot (*Daucus carota L.*) materials based on multidimensional quality traits.

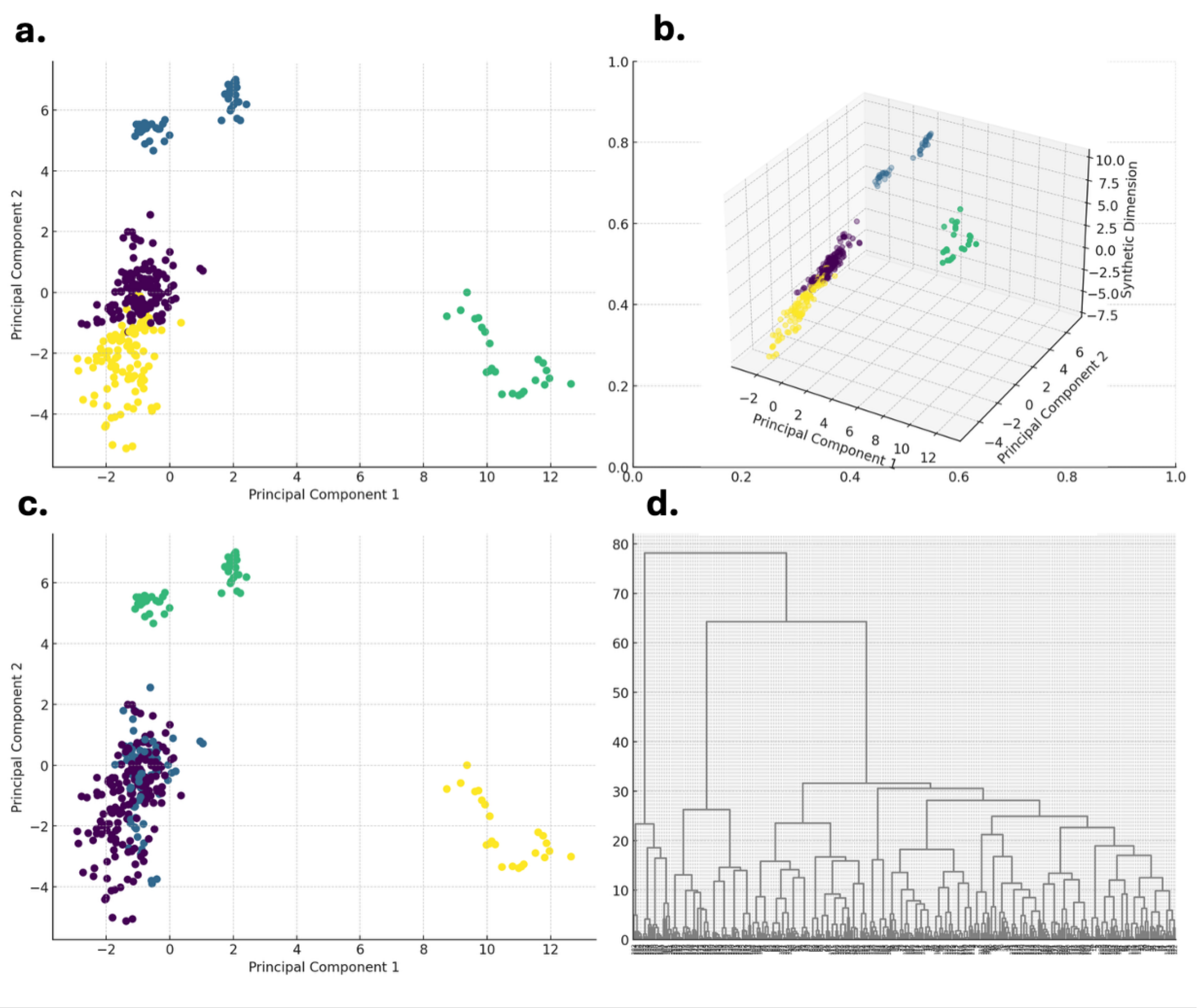

Supplementary Figure 2. *Comparison of clustering approaches for carrot (Daucus carota L.) materials based on multidimensional quality traits.*(a) PCA score plot (PC1 vs PC2) showing sample distribution in reduced dimensional space. (b) Three-dimensional PCA score plot (PC1 vs PC2 vs PC3) visualizing the multivariate structure of the dataset. (c) K-means clustering solution (k = 4) projected onto the PC1–PC2 space, highlighting cluster partitioning.(d) Agglomerative hierarchical clustering dendrogram using Ward’s method, illustrating hierarchical relationships among samples based on the same set of standardized variables.

Code	Modal root	Root color	Carrot type	Main agronomic and morphological descriptors	Crop cycle
**1NAN**	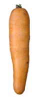	Orange	Nantes	Cylindrical to slightly tapered root; smooth epidermis; high external–internal uniformity.	100–140 days
**2CHAN**	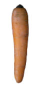	Orange	Chantenay	Short to medium root; broad-shouldered architecture; compact conical profile.	100–120 days
**3CHAN**	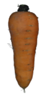	Orange	Chantenay	Conical root; wide basal diameter; regular commercial length.	120 days
**4CHAN**	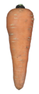	Orange	Chantenay	Compact root architecture; broad upper section; moderate distal tapering.	135–160 days
**5BER**	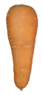	Orange	Berlicum	Elongated cylindrical root; straight longitudinal axis; moderate basal diameter.	120–150 days
**6KUR**	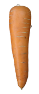	Orange	Kuroda	Conical root; pronounced shoulder; progressive tapering toward the distal end.	110–130 days
**7NAN**	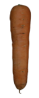	Orange	Nantes	Cylindrical root; elongated profile; uniform caliber along the longitudinal axis.	120–140 days
**8NAN**	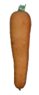	Orange	Nantes	Cylindrical root; regular surface; high shape uniformity.	110 days
**9NAN**	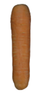	Orange	Nantes	Elongated cylindrical root; regular contour; moderate tapering.	120 days
**10BER**	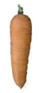	Orange	Berlicum	Long cylindrical root; straight profile; large commercial caliber.	115–120 days
**11NAN**	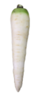	White	Nantes	Cylindrical root; smooth surface; narrow to moderate basal diameter.	110–120 days
**12NAN**	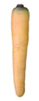	Yellow	Nantes	Cylindrical root; smooth epidermis; narrow to moderate basal diameter.	110–120 days
**13FLA**	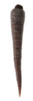	Purple	Flakkee	Cylindrical root; elongated profile; moderate basal diameter.	100–120 days
**14BER**	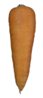	Orange	Berlicum	Long cylindrical root; straight longitudinal axis; large commercial caliber.	125–135 days

Supplementary Figure 3. Comparative profile of the fourteen carrot materials used for multidimensional quality characterization and industrial suitability assessment.

Supplementary Material

Supplementary Table 1. *Tukey's HSD multiple-comparison grouping of multidimensional quality traits across carrot (Daucus carota L.) varieties and multivariate clusters.*

	Cluster 1							Cluster 2		Cluster 3	Cluster 4				Cluster 1	Cluster 2	Cluster 3	Cluster 4
Variable	2CHAN	3CHAN	7NAN	8NAN	9NAN	10BER	14BER	11NAN	12NAN	13FLA	1NAN	4CHAN	5BER	6KUR				
pH	6.22 ± 0.20 b	6.19 ± 0.09 c	6.29 ± 0.15 b	6.25 ± 0.08 b	6.32 ± 0.15 b	6.07 ± 0.19 c	6.16 ± 0.31 c	5.91 ± 0.04 d	6.03 ± 0.12 d	5.99 ± 0.08 d	6.34 ± 0.05 b	6.41 ± 0.09 a	6.50 ± 0.12 a	6.42 ± 0.11 a	6.23 ± 0.18 B	5.97 ± 0.11 C	5.99 ± 0.08 C	6.42 ± 0.12 A
Brix	7.76 ± 0.30 b	7.11 ± 0.44 c	7.59 ± 0.48 b	7.99 ± 0.68 b	7.64 ± 0.45 b	7.98 ± 0.52 b	7.15 ± 0.60 c	6.77 ± 0.34 c	6.83 ± 0.34 c	8.52 ± 0.77 a	7.95 ± 0.11 b	7.65 ± 0.40 b	7.77 ± 0.65 b	8.75 ± 0.34 a	7.79 ± 0.52 C	6.80 ± 0.34 D	8.52 ± 0.77 A	8.05 ± 0.60 B
Dry_matter	10.32 ± 0.29 c	9.89 ± 0.62 d	10.26 ± 0.42 c	10.91 ± 0.23 b	10.49 ± 0.31 c	10.92 ± 1.14 b	10.65 ± 0.77 b	9.16 ± 0.46 e	8.94 ± 0.31 e	11.64 ± 0.82 a	10.65 ± 0.21 b	11.64 ± 0.74 a	11.11 ± 0.38 b	11.85 ± 0.79 a	10.58 ± 0.64 B	9.05 ± 0.40 C	11.64 ± 0.82 A	11.29 ± 0.74 A
L_C (External color)	56.56 ± 1.92 c	57.95 ± 1.78 b	55.55 ± 1.67 c	57.49 ± 1.98 b	55.96 ± 1.84 c	57.24 ± 2.02 b	58.56 ± 1.56 b	63.99 ± 2.00 a	63.08 ± 2.83 a	25.27 ± 2.12 d	57.80 ± 1.87 b	57.21 ± 2.05 b	57.03 ± 1.24 b	58.71 ± 1.71 b	56.56 ± 2.00 C	63.54 ± 2.47 A	25.27 ± 2.12 D	57.72 ± 1.82 B
a_C (External color)	21.03 ± 1.60 b	22.16 ± 2.05 a	19.84 ± 1.32 b	21.65 ± 2.88 a	19.27 ± 1.61 b	20.09 ± 2.07 b	22.83 ± 1.57 a	-1.93 ± 0.36 e	0.82 ± 0.89 d	6.79 ± 1.77 c	21.91 ± 1.94 a	23.05 ± 3.81 a	22.33 ± 1.22 a	23.13 ± 2.16 a	20.38 ± 2.12 B	-0.56 ± 1.55 D	6.79 ± 1.77 C	22.58 ± 2.37 A

**b_C (External color)**	37.50 ± 4.42 b	41.94 ± 5.01 a	33.51 ± 2.72 c	35.38 ± 5.17 b	34.61 ± 3.84 b	32.72 ± 2.79 c	35.96 ± 3.28 b	11.88 ± 1.41 d	41.20 ± 2.84 a	1.23 ± 0.47 e	34.85 ± 4.88 b	38.64 ± 4.05 a	37.91 ± 2.66 b	36.69 ± 2.87 b	34.74 ± 4.18 A	26.54 ± 14.98 B	1.23 ± 0.47 C	36.91 ± 3.91 A
**C_C (External color)**	43.01 ± 4.59 b	47.46 ± 5.29 a	38.96 ± 2.90 c	41.49 ± 5.86 b	39.64 ± 4.01 b	38.42 ± 3.33 c	42.61 ± 3.54 b	12.04 ± 1.41 d	41.23 ± 2.83 b	6.94 ± 1.72 e	41.18 ± 5.13 b	45.03 ± 5.33 a	44.01 ± 2.82 a	43.42 ± 3.23 b	40.30 ± 4.53 B	26.63 ± 14.91 C	6.94 ± 1.72 D	43.30 ± 4.35 A
**h_C (External color)**	60.55 ± 1.35 c	62.02 ± 1.47 c	59.32 ± 1.24 d	58.51 ± 1.04 d	60.71 ± 1.57 c	58.48 ± 1.40 d	57.50 ± 1.11 d	99.33 ± 1.72 a	88.75 ± 1.36 b	11.30 ± 5.62 e	57.59 ± 1.55 d	59.40 ± 2.07 d	59.45 ± 1.00 d	57.79 ± 2.09 d	59.51 ± 1.62 B	94.04 ± 5.56 A	11.30 ± 5.62 C	58.50 ± 1.89 B
**L_I (Internal color)**	59.79 ± 5.48 b	54.28 ± 4.39 c	61.17 ± 3.87 b	56.22 ± 3.49 c	57.49 ± 4.18 c	52.77 ± 6.78 c	54.01 ± 5.23 c	65.56 ± 6.43 a	69.56 ± 4.72 a	35.99 ± 8.59 d	54.81 ± 4.76 c	63.48 ± 3.08 b	55.60 ± 5.09 c	55.20 ± 4.16 c	57.49 ± 5.65 B	67.56 ± 5.93 A	35.99 ± 8.59 C	56.86 ± 5.48 B
**a_I (Internal color)**	20.65 ± 1.62 b	16.48 ± 4.95 c	24.43 ± 4.41 a	25.05 ± 3.68 a	23.36 ± 3.26 a	18.87 ± 6.55 b	20.67 ± 4.54 b	-3.54 ± 0.49 d	-3.95 ± 2.44 d	20.26 ± 5.64 b	22.35 ± 4.73 b	25.49 ± 2.23 a	20.68 ± 4.34 b	27.03 ± 4.84 a	22.47 ± 4.77 AB	-3.74 ± 1.75 C	20.26 ± 5.64 B	23.78 ± 4.93 A
**b_I (Internal color)**	42.50 ± 3.75 b	37.63 ± 6.62 b	49.49 ± 5.93 a	44.81 ± 6.25 a	45.91 ± 4.32 a	39.17 ± 9.54 b	41.22 ± 7.96 b	14.94 ± 1.93 c	45.49 ± 4.53 a	5.63 ± 4.08 d	41.89 ± 7.55 b	50.22 ± 5.22 a	41.22 ± 7.62 b	46.40 ± 7.00 a	44.38 ± 7.09 A	30.22 ± 15.82 B	5.63 ± 4.08 C	44.58 ± 7.74 A
**C_I (Internal color)**	47.28 ± 3.87 b	41.19 ± 7.92 b	55.23 ± 7.19 a	51.35 ± 7.19 a	51.57 ± 5.06 a	43.53 ± 11.43 b	46.12 ± 9.13 b	15.36 ± 1.96 c	45.74 ± 4.47 b	21.90 ± 4.57 c	47.49 ± 8.85 b	56.40 ± 5.14 a	46.15 ± 8.68 b	53.73 ± 8.40 a	49.79 ± 8.33 A	30.55 ± 15.73 B	21.90 ± 4.57 C	50.58 ± 8.97 A
**h_I (Internal color)**	64.01 ± 1.63 c	67.15 ± 3.53 c	63.98 ± 1.86 c	60.87 ± 1.11 d	63.16 ± 2.16 c	65.00 ± 2.31 c	63.53 ± 1.00 c	103.41 ± 1.49 a	95.18 ± 3.09 b	17.56 ± 14.49 e	62.07 ± 1.33 d	62.97 ± 2.51 c	63.56 ± 1.56 c	59.91 ± 1.47 d	63.40 ± 2.31 B	99.29 ± 4.80 A	17.56 ± 14.49 C	62.07 ± 2.21 B

**Carrot_weight**	170.26 ± 42.50 a	140.13 ± 55.75 b	156.28 ± 37.21 b	186.31 ± 33.94 a	200.82 ± 40.44 a	195.64 ± 55.58 a	183.04 ± 49.67 a	164.49 ± 48.99 a	190.10 ± 56.82 a	98.56 ± 24.41 c	154.13 ± 23.17 b	143.77 ± 25.89 b	184.35 ± 54.68 a	146.12 ± 42.67 b	181.86 ± 45.01 A	177.29 ± 54.06 AB	98.56 ± 24.41 C	157.98 ± 42.19 B
**Carrot_length**	22.55 ± 8.79 a	13.08 ± 1.40 c	17.77 ± 1.43 b	17.89 ± 1.31 b	18.09 ± 1.70 b	19.10 ± 2.31 b	16.99 ± 1.86 b	19.52 ± 2.05 b	23.00 ± 2.77 a	20.40 ± 2.46 a	18.52 ± 2.07 b	16.00 ± 2.76 c	17.17 ± 1.73 b	16.84 ± 1.75 b	19.08 ± 4.53 B	21.26 ± 2.98 A	20.40 ± 2.46 AB	17.21 ± 2.22 C
**Mean_diameter**	4.26 ± 0.43 b	4.67 ± 0.65 a	4.24 ± 0.46 b	4.58 ± 0.41 a	4.61 ± 0.44 a	4.52 ± 0.39 a	4.47 ± 0.46 a	4.20 ± 0.39 b	3.93 ± 0.39 b	3.81 ± 0.43 c	4.30 ± 0.25 a	4.31 ± 0.28 a	4.70 ± 0.52 a	4.31 ± 0.42 a	4.44 ± 0.45 A	4.06 ± 0.41 B	3.81 ± 0.43 B	4.41 ± 0.42 A
**aerobic_mesophilic**	25610 ± 40391 b	73950 ± 98378 b	251900 ± 388602 a	86225 ± 137904 b	39852 ± 43820 b	21710 ± 16966 b	13847 ± 10954 b	168075 ± 284333 a	4175 ± 1314 b	172850 ± 281487 a	16075 ± 12352 b	34266 ± 24085 b	64500 ± 47307 b	27575 ± 16267 b	85059 ± 202863 AB	86125 ± 215458 AB	172850 ± 281487 A	35693 ± 33804 B
**Total_Coliforms**	3862 ± 6571 b	10057 ± 15353b	173982 ± 292594 a	111222 ± 193913 a	6392 ± 5505 b	3977 ± 5923 b	1860 ± 1343 b	74737 ± 77123b	2104 ± 883 b	53225 ± 44685 b	2277 ± 1706 b	12212 ± 8794 b	22050 ± 25238 b	4800 ± 4559 b	59887 ± 169802 A	38421 ± 65253 AB	53225.00 ± 44685.14 AB	10209 ± 15779 B
**Moisture**	89.68 ± 0.29 b	90.11 ± 0.62 b	89.74 ± 0.42 b	89.09 ± 0.23 c	89.51 ± 0.31 c	89.08 ± 1.14 c	89.35 ± 0.77 c	90.84 ± 0.46 a	91.06 ± 0.31 a	88.36 ± 0.82 d	89.35 ± 0.21 c	88.36 ± 0.74 d	88.89 ± 0.38 d	88.15 ± 0.79 e	89.42 ± 0.64 B	90.95 ± 0.40 A	88.36 ± 0.82 C	88.71 ± 0.74 C
**Total_sugars**	3.54 ± 0.97 b	3.81 ± 0.90 a	3.51 ± 0.70 b	3.24 ± 0.74 b	3.47 ± 0.48 b	3.73 ± 0.34 a	3.79 ± 1.26 a	3.24 ± 0.15 b	3.05 ± 0.65 b	3.68 ± 0.70 b	4.28 ± 1.15 a	4.40 ± 0.34 a	4.51 ± 0.91 a	3.67 ± 1.32 b	3.50 ± 0.69 BC	3.15 ± 0.48 C	3.68 ± 0.70 B	4.20 ± 1.07 A

**Ash**	1.01 ± 0.14 b	1.19 ± 0.10 a	1.16 ± 0.28 a	1.00 ± 0.12 b	0.91 ± 0.05 c	0.92 ± 0.17 c	1.13 ± 0.06 a	1.06 ± 0.16 b	1.09 ± 0.14 a	1.20 ± 0.11 a	1.00 ± 0.08 b	1.20 ± 0.20 a	1.04 ± 0.14 b	1.08 ± 0.10 a	1.00 ± 0.19 C	1.07 ± 0.15 B	1.20 ± 0.11 A	1.07 ± 0.15 B
**Fe**	0.21 ± 0.09 c	0.27 ± 0.11 c	0.40 ± 0.13 b	0.36 ± 0.11 b	0.44 ± 0.18 b	0.23 ± 0.04 c	0.29 ± 0.08 c	0.26 ± 0.11 c	0.35 ± 0.18 b	0.69 ± 0.52 a	0.25 ± 0.01 c	0.53 ± 0.29 a	0.48 ± 0.17 b	0.22 ± 0.08 c	0.33 ± 0.15 B	0.31 ± 0.16 B	0.69 ± 0.52 A	0.36 ± 0.21 B
**Na**	9.57 ± 1.91 b	8.32 ± 4.46 b	11.33 ± 1.04 a	7.69 ± 2.35 b	6.48 ± 0.35 c	12.59 ± 2.82 a	8.75 ± 1.87 b	6.88 ± 0.82 c	9.71 ± 3.18 b	4.90 ± 2.28 c	7.69 ± 3.40 b	9.65 ± 1.11 b	10.45 ± 2.93 a	8.15 ± 3.61 b	9.53 ± 2.94 A	8.29 ± 2.71 A	4.90 ± 2.28 B	8.94 ± 3.18 A
**Ca**	34.25 ± 10.64 b	46.19 ± 12.44 a	47.12 ± 15.76 a	38.67 ± 10.71 a	38.37 ± 10.02 a	31.25 ± 14.45 b	45.46 ± 13.14 a	35.29 ± 14.25 b	38.22 ± 16.67 a	48.21 ± 14.33 a	38.95 ± 9.57 a	48.31 ± 14.22 a	47.63 ± 11.95 a	31.46 ± 10.89 b	37.93 ± 13.44 B	36.75 ± 15.41 B	48.21 ± 14.33 A	41.14 ± 13.35 AB
**Cu**	0.49 ± 0.11 b	0.52 ± 0.17 a	0.55 ± 0.19 a	0.38 ± 0.14 b	0.43 ± 0.17 b	0.46 ± 0.09 b	0.41 ± 0.12 b	0.56 ± 0.10 a	0.52 ± 0.07 a	0.54 ± 0.08 a	0.63 ± 0.09 a	0.57 ± 0.19 a	0.50 ± 0.18 b	0.56 ± 0.11 a	0.46 ± 0.15 B	0.54 ± 0.09 A	0.54 ± 0.08 A	0.57 ± 0.15 A
**Ni**	0.21 ± 0.13 b	0.19 ± 0.07 b	0.34 ± 0.27 a	0.17 ± 0.17 b	0.17 ± 0.14 b	0.21 ± 0.12 b	0.32 ± 0.09 a	0.17 ± 0.10 b	0.15 ± 0.04 b	0.43 ± 0.21 a	0.16 ± 0.02 b	0.33 ± 0.20 a	0.20 ± 0.15 b	0.24 ± 0.18 b	0.22 ± 0.18 B	0.16 ± 0.08 B	0.43 ± 0.21 A	0.23 ± 0.16 B
**Total_fiber**	2.33 ± 0.46 c	3.27 ± 0.61 b	2.52 ± 0.53 c	2.96 ± 0.51 b	2.96 ± 0.86 b	2.48 ± 0.77 c	3.24 ± 0.53 b	2.43 ± 0.52 c	2.82 ± 0.73 b	4.15 ± 1.06 a	2.79 ± 1.50 b	3.17 ± 0.53 b	2.15 ± 0.65 c	2.50 ± 0.80 c	2.65 ± 0.69 B	2.63 ± 0.65 B	4.15 ± 1.06 A	2.62 ± 1.02 B
**Soluble_fiber**	0.23 ± 0.16 c	0.34 ± 0.51 b	0.00 ± 0.00 c	0.70 ± 0.41 a	0.95 ± 0.16 a	0.52 ± 0.15 b	0.00 ± 0.00 c	0.42 ± 0.26 b	0.73 ± 0.36 a	0.89 ± 0.46 a	0.23 ± 0.14 c	0.31 ± 0.22 b	0.00 ± 0.00 c	0.00 ± 0.00 c	0.48 ± 0.40 B	0.58 ± 0.34 B	0.89 ± 0.46 A	0.12 ± 0.18 C

Supplementary Material

**Insoluble_fiber**	2.18 ± 0.45 b	2.93 ± 0.60 b	2.22 ± 0.48 b	2.47 ± 0.23 b	3.11 ± 1.20 a	2.23 ± 0.51 b	3.77 ± 1.80 a	2.08 ± 0.30 c	2.48 ± 0.33 b	3.08 ± 0.60 a	2.63 ± 1.43 b	2.86 ± 0.33 b	2.09 ± 0.62 c	2.19 ± 0.45 b	2.44 ± 0.74 B	2.28 ± 0.37 B	3.08 ± 0.60 A	2.42 ± 0.89 B
**Protein**	0.84 ± 0.11 c	1.03 ± 0.18 b	1.10 ± 0.37 b	0.13 ± 0.23 e	0.69 ± 0.05 d	0.73 ± 0.16 d	0.91 ± 0.07 c	0.98 ± 0.08 b	1.07 ± 0.14 b	1.41 ± 0.16 a	0.96 ± 0.08 b	1.12 ± 0.12 b	0.90 ± 0.13 c	0.78 ± 0.05 c	0.70 ± 0.38 C	1.02 ± 0.12 B	1.41 ± 0.16 A	0.93 ± 0.16 B
**Carbohydrates**	8.87 ± 1.31 a	7.64 ± 0.83 b	7.76 ± 0.81 b	9.04 ± 1.49 a	8.34 ± 1.09 b	8.96 ± 1.89 a	8.26 ± 1.12 b	6.91 ± 0.88 c	6.73 ± 0.60 c	9.78 ± 0.48 a	7.09 ± 3.58 b	10.22 ± 0.47 a	9.66 ± 0.22 a	9.82 ± 0.48 a	8.59 ± 1.43 B	6.82 ± 0.75 C	9.78 ± 0.48 A	9.13 ± 2.23 AB
**Calories**	34.43 ± 3.28 b	27.45 ± 3.12 c	30.35 ± 2.75 c	32.00 ± 4.65 b	29.70 ± 3.50 c	33.85 ± 6.70 b	29.30 ± 4.64 c	26.50 ± 2.87 d	26.78 ± 1.35 c	38.25 ± 1.92 a	32.40 ± 3.21 b	39.40 ± 3.51 a	37.95 ± 1.90 a	39.57 ± 4.85 a	32.07 ± 4.72 B	26.64 ± 2.22 C	38.25 ± 1.92 A	37.19 ± 4.57 A
**Total_carotenes**	1.44 ± 0.70 b	1.41 ± 0.26 b	1.47 ± 0.21 b	1.55 ± 0.53 b	1.32 ± 0.23 b	1.55 ± 0.91 b	1.65 ± 0.79 b	0.00 ± 0.00 c	0.15 ± 0.10 c	0.13 ± 0.06 c	2.07 ± 1.12 a	1.42 ± 0.33 b	2.12 ± 0.28 a	2.42 ± 0.75 a	1.47 ± 0.58 B	0.08 ± 0.10 C	0.13 ± 0.06 C	2.05 ± 0.79 A
**B_Carotene**	0.30 ± 0.06 c	0.30 ± 0.02 c	0.35 ± 0.01 b	0.40 ± 0.03 a	0.34 ± 0.04 b	0.35 ± 0.03 b	0.39 ± 0.02 a	0.00 ± 0.00 d	0.00 ± 0.00 d	0.00 ± 0.00 d	0.30 ± 0.02 c	0.33 ± 0.03 b	0.31 ± 0.03 c	0.40 ± 0.04 a	0.35 ± 0.05 A	0.00 ± 0.00 B	0.00 ± 0.00 B	0.33 ± 0.05 A
**TPC**	94.37 ± 35.20 c	93.57 ± 38.84 c	126.64 ± 60.78 b	126.46 ± 56.38 b	86.06 ± 26.48 c	97.01 ± 47.20 c	110.14 ± 46.86 c	194.24 ± 122.28 b	127.59 ± 43.75 b	683.83 ± 191.60 a	82.51 ± 24.92. c	82.48 ± 27.56. c	64.44 ± 17.46 c	77.84 ± 36.28 c	106.11 ± 49.28 C	160.92 ± 96.89 B	683.83 ± 191.60 A	76.44 ± 27.95. D
**FRAP**	251.74 ± 47.53 b	232.91 ± 61.25 b	261.00 ± 111.61 b	299.96 ± 43.05 b	353.77 ± 96.70 b	311.61 ± 136.18 b	351.18 ± 51.66 b	503.75 ± 92.42	440.51 ± 82.88 b	3118.54 ± 1639.97 a	256.66 ± 101.47 b	261.26 ± 71.15 b	239.41 ± 43.46 b	290.44 ± 122.69 b	295.61 ± 99.80 B	472.13 ± 92.53 B	3118.54 ± 1639.97 A	261.99 ± 91.48 B

								b										
**DPPH**	235.10 ± 103.25 c	257.82 ± 110.82 c	404.44 ± 262.05 b	413.46 ± 278.48 b	366.74 ± 275.83 b	396.44 ± 317.55 b	130.85 ± 74.36 c	651.44 ± 716.38 b	248.89 ± 121.34 c	1830.45 ± 502.25 a	268.66 ± 111.49 c	200.17 ± 103.47 c	345.10 ± 257.14 c	378.52 ± 360.88 b	363.24 ± 262.50 B	450.17 ± 547.47 B	1830.45 ± 502.25 A	304.64 ± 245.91 B
**ORAC_H**	3179.81 ± 839.25 b	3218.06 ± 574.74 b	4067.89 ± 1291.35 b	2716.28 ± 545.12 b	2695.38 ± 844.57 b	2780.83 ± 572.05 b	2064.76 ± 618.77 c	3145.50 ± 1040.26 b	2719.65 ± 892.27 b	20248.62 ± 5703.06 a	3657.03 ± 1248.56 b	2928.39 ± 390.93 b	2930.78 ± 907.18 b	3080.04 ± 1161.32 b	3088.04 ± 995.15 B	2932.58 ± 982.58 B	20248.62 ± 5703.06 A	3163.77 ± 1042.40 B

*Values are expressed as mean ± standard deviation (SD). Different lowercase letters indicate significant differences among carrot varieties according to Tukey's HSD test (α = 0.05). Different uppercase letters indicate significant differences among clusters generated at the multivariate level according to Tukey's HSD test (α = 0.05).

Supplementary Table 2. Regulations applicable to carrots for Fresh Consumption, Processed or Functional Foods, Pet Feed, and Natural Cosmetics

Fresh Consumption			
**Food Safety Target**	**Applicable Regulations**	**Reference Limits Required (when applicable)**	**Key Aspects**
Process hygiene and compliance with Maximum Residue Limits (MRLs)	Codex Alimentarius (CXC 1; CXC 53) – Code of Hygienic Practice for Fresh Fruits and Vegetables	Microbiology: No mandatory microbiological limits established for fresh vegetables; E. coli recommended as an indicator organism (typically $<10^2–10^3$ CFU/g as an operational hygiene criterion). Pesticides: Codex Maximum Residue Limits (CXL). MRLs according to Codex for carrots apply for the following pesticides: Carbaryl 0.5 mg/kg, Carbendazim 0.2 mg/kg, Chlorantraniliprole 0.08 mg/kg, Cycloxydim 5 mg/kg, Cyprodinil 1.5 mg/kg, DDT 0.2 mg/kg, Deltamethrin 0.02 mg/kg, Difenoconazole 0.2 mg/kg, Dithiocarbamates 1 mg/kg, Fenamidone 0.2 mg/kg, Fluazaindolizine 0.4 mg/kg, Fluazifop-p-butyl 0.6 mg/kg, Fludioxonil 1 mg/kg, Fluensulfone 4 mg/kg, Fluopyram 0.4 mg/kg, Fluxapyroxad 1 mg/kg, Glufosinate-ammonium 0.05 mg/kg, Isopyrazam 0.15 mg/kg, Metalaxyl 0.02 mg/kg, Methoxyfenozide 0.5 mg/kg, Oxamyl 0.01 mg/kg, Pendimethalin 0.5 mg/kg, Penthiopyrad 0.6 mg/kg, Permethrin 0.1 mg/kg, Pyrimethanil 1 mg/kg, Spirotetramat 0.04 mg/kg, Sulfoxaflor 0.05 mg/kg, Tebuconazole 0.4 mg/kg, Trifloxystrobin 0.1 mg/kg.	Preventive approach (GAP/GHP); verification through indicators; enforceable MRLs for trade; emphasis on agricultural water, soil management, and harvest, postharvest hygiene.
	EU Regulation (EC) No. 396/2005 establishing pesticide MRLs in food and feed;<br><br>Regulation (EC) No. 2073/2005	Pesticides: MRLs by active ingredient (e.g., acetamiprid 0.3 mg/kg; boscalid 1.5 mg/kg; values vary by active ingredient).<br><br>Microbiology: Chopped fruits and vegetables (ready to eat) and unpasteurized fruit and vegetable juices (ready to eat). E. coli minimum 100 CFU/g (ISO 16649-1 or 2 standard). Salmonella: Absent in 25 g (ISO 6579)	EU harmonization; practice-based control; auditable MRLs.
	FSMA Produce Safety Rule (21 CFR 112)	Microbiology: No microbiological limits; agricultural water with E. coli ≤ 126 CFU/100 mL (historical water quality criterion; subject to current regulation). Pesticides: EPA tolerances by active ingredient/crop.	Control of water, soil, and hygiene.

	Colombia (ICA / INVIMA / NTC)	Harmonized reference microbiological criteria used: Salmonella spp.: absence in 25 g; Listeria monocytogenes: absence in 25 g;E. coli $\leq 10^2$–10^3 CFU/g (operational hygiene criterion).<br><br>*Pesticides: MRLs adopted and harmonized with Codex and international regulations.	Preventive approach and traceability for foods intended for consumption.
		**Processed or Functional Foods**	
Food Safety Target	Applicable Regulations	Reference Limits Required	Key Aspects
Pathogen control and process validation	Codex Alimentarius (HACCP; RTE foods)	Salmonella spp.: absence in 25 g. Listeria monocytogenes: according to product category/shelf life (Codex guidance).	Validated Hazard Analysis and Critical Control Points (HACCP); batch verification.
	EU Regulation (EC) No. 2073/2005	Listeria monocytogenes: ≤100 CFU/g during shelf life (if growth is not supported) or absence in 25 g (if growth is supported). Salmonella: absence in 25 g.	Mandatory RTE criteria.
	EU Regulation (EC) No. 396/2005	Pesticides: MRLs applicable to the ingredient; use of processing factors where relevant.	Compliance at raw material and finished product levels.
	Colombia (Decree 3075/1997; INVIMA)	RTE pathogens: absence of Salmonella; limits for L. monocytogenes according to category. Pesticides: current MRLs.	GMP + official surveillance.
		**Pet Feed**	
Food Safety Target	Applicable Regulations	Reference Limits Required	Key Aspects

Prevention of hazard transfer	Codex (CAC/RCP 54-2004)	Microbiology: No universal limits; process-based control. Chemicals: Risk assessment and compliance with applicable limits.	Risk-based approach.
	EU Directive 2002/32/EC; Regulation (EC) No. 767/2009	Metals (feed): Pb ≤ 10 mg/kg; Cd ≤ 1 mg/kg (plant-based feed materials; EU reference values).	Emphasis on contaminants and stability.
	Colombia (ICA)	Chemicals: Compliance with applicable limits according to species/use.	Registration, traceability, moisture and storage control.
		**Natural Cosmetics**	
Safety Target	Applicable Regulations	Reference Limits Required	Key Aspects
Ingredient and finished product safety	EU Regulation (EC) No. 1223/2009	Microbiology: According to ISO 17516 (see below). Chemicals: Toxicological evaluation (no food MRLs).	Mandatory safety dossier.
	ISO 17516	Category 1 products (children, eye area, mucous membranes): TAMC $\leq 10^2$ CFU/g; TYMC $\leq 10^1$ CFU/g; absence of S. aureus, P. aeruginosa, C. albicans.Category 2 products: TAMC $\leq 10^3$ CFU/g; TYMC $\leq 10^2$ CFU/g; same pathogens absent.	Finished product limits.
	Colombia (INVIMA)	Microbiology: Aligned with ISO 17516. Chemicals: Case-by-case evaluation.	
